# Supplementary material for: Synthesis and Effect of Conformationally Locked Carbocyclic Guanine Nucleotides on Dynamin
Source: Biomolecules. 2022 Apr 16;12(4):584. doi: 10.3390/biom12040584 (PMC9031165; doi:10.3390/biom12040584)
Supplement: Supplementary file 1 [file biomolecules-12-00584-s001.zip › biomolecules-1664002-supplementary.pdf]

Supplementary information

Synthesis and Effect of Conformationally Locked Carbocyclic Guanine Nucleotides on Dynamin

Kiran S. Toti[1], John R. Jimah[2], Veronica Salmaso[1], Jenny E. Hinshaw[2], Kenneth A. Jacobson[1]

[1]Laboratory of Bioorganic Chemistry, National Institute of Diabetes and Digestive and Kidney Diseases, NIH, Bethesda, MD, USA.

[2]Laboratory of Cell and Molecular Biology, National Institute of Diabetes and Digestive and Kidney Diseases, NIH, Bethesda, MD, USA.

Table of Contents

Supplementary Figure S1 — Page S1
Supplementary Figure S2 — Page S2
[1]H, ^{31}P, HRMS and HPLC Copies of Final Compounds — Page S4-22
[1]H, ^{31}P, and HRMS Copies of Intermediates — Page S23-33

Supplementary figure S1

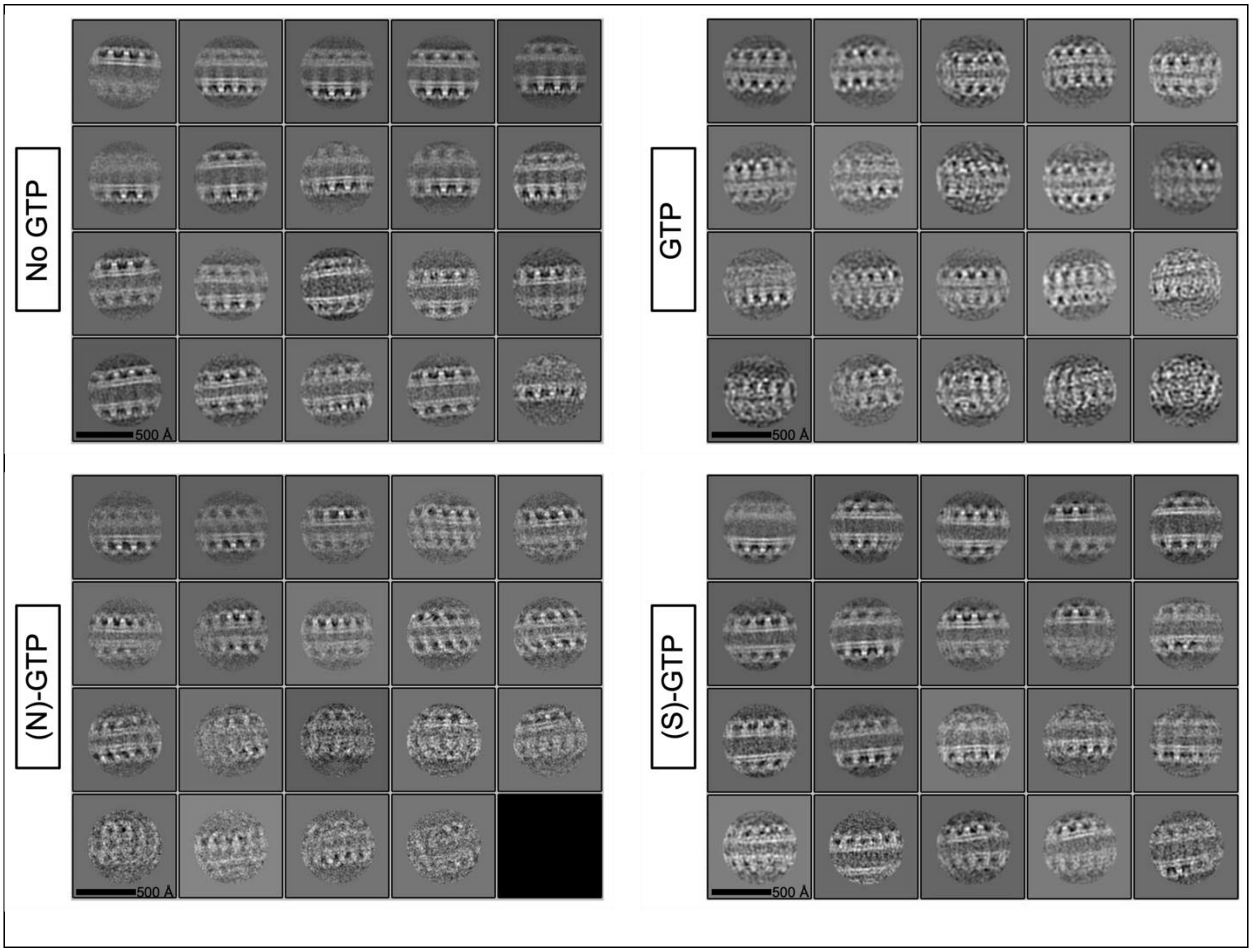

Supplementary Figure S1. 2D classes of cryoEM micrographs of dynamin-decorated lipid tubules without nucleotide or after treatment with GTP (Jena Biosciences Co), (N)-GTP, or (S)-GTP.

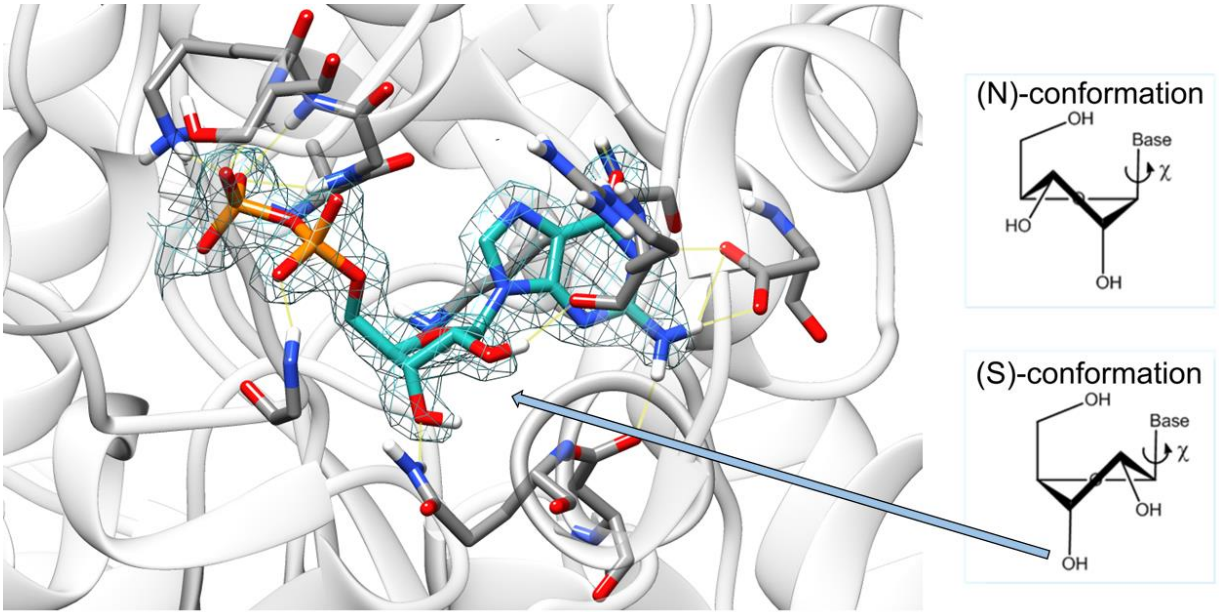

Supplementary Figure S2. Atomic model of dynamin (PDB ID: 2X2E) bound to GDP in South (S)- conformation with electron density shown around the ligand. Right panel shows the north and south conformations, with the south conformation in agreement with the electron density around the nucleotide in the left panel. The image was generated using the software UCSF Chimera (Pettersen, E.F., Goddard, T.D., Huang, C.C., Couch, G.S., Greenblatt, D.M., Meng, E.C., and Ferrin, T.E. UCSF Chimera - A Visualization System for Exploratory Research and Analysis. J. Comput. Chem. 25:1605-1612 (2004).). Selected protein residues interacting with the nucleotide (cyan) are shown in grey sticks. H-bonds were predicted after addition of hydrogens and are shown by yellow lines.

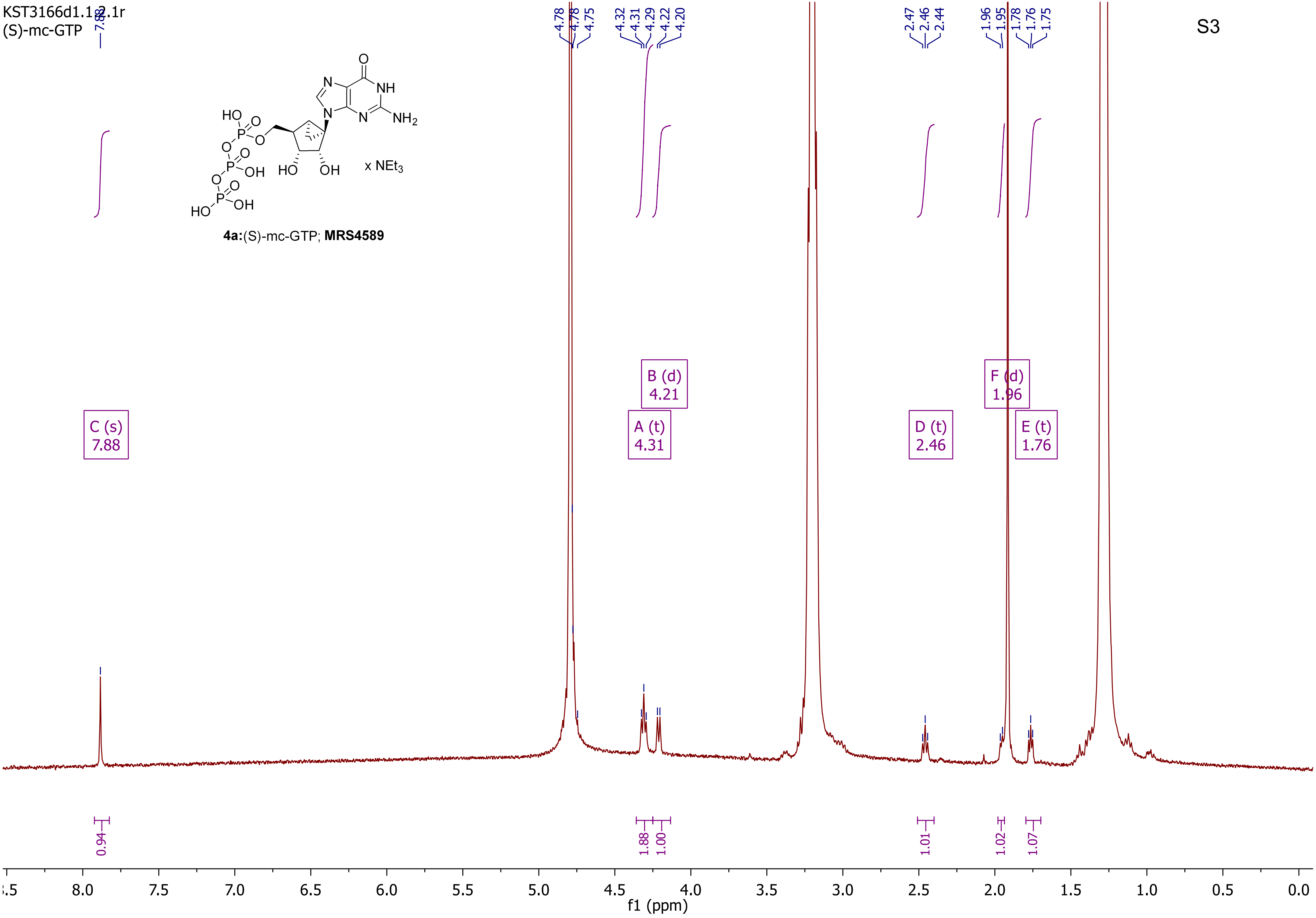

4a:(S)-mc-GTP; **MRS4589**

KST3166d1-p31.1.2.1r
(S)-mc-GTP

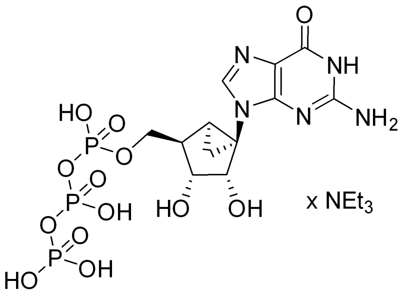

4a:(S)-mc-GTP; **MRS4589**

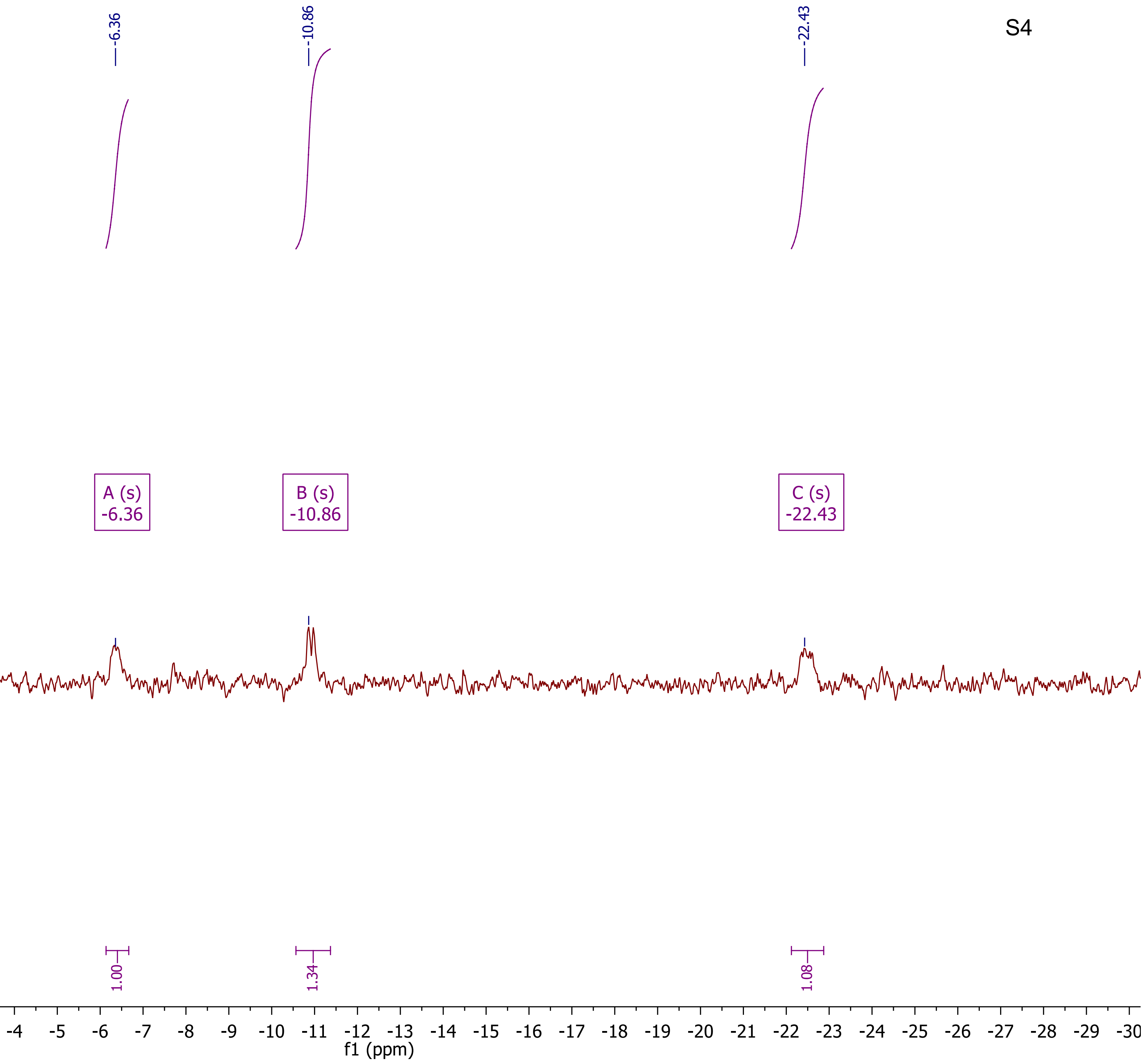

Elemental Composition Report

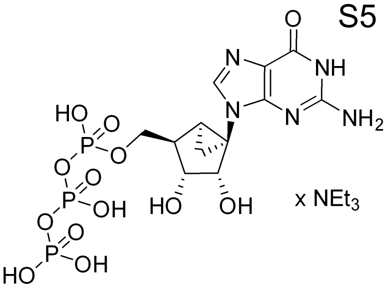

4a:(S)-mc-GTP; **MRS4589**

Single Mass Analysis

Tolerance = 10.0 mDa / DBE: min = -1.5, max = 100.0
Element prediction: Off
Number of isotope peaks used for i-FIT = 3

Monoisotopic Mass, Even Electron Ions
89 formula(e) evaluated with 5 results within limits (up to 50 closest results for each mass)
Elements Used:
C: 0-200 H: 0-200 N: 5-5 O: 0-60 P: 3-3
KST-12OCT18-3-166-D1 221 (3.755) AM2 (Ar,22000.0,0.00,0.00); ABS
TOF MS ES-

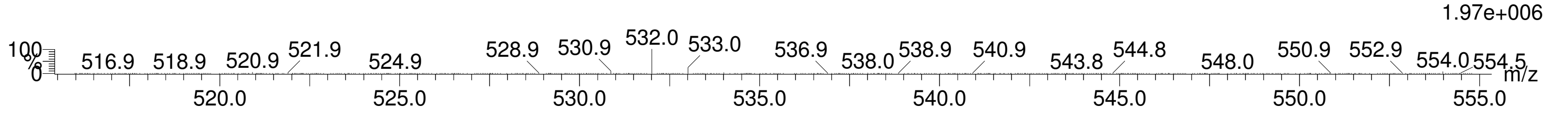

Minimum:				-1.5				
Maximum:		10.0	5.0	100.0				
Mass	Calc. Mass	mDa	PPM	DBE	i-FIT	Norm	Conf(%)	Formula
532.0040	532.0036	0.4	0.8	8.5	354.7	1.878	15.29	C12 H17 N5 O13 P3
	532.0071	-3.1	-5.8	30.5	356.5	3.676	2.53	C30 H9 N5 P3
	532.0094	-5.4	-10.2	-0.5	359.1	6.248	0.19	C5 H21 N5 O18 P3
	531.9977	6.3	11.8	17.5	353.2	0.313	73.10	C19 H13 N5 O8 P3
	532.0130	-9.0	-16.9	21.5	355.3	2.421	8.89	C23 H13 N5 O5 P3

Data File C:\HPCHEM\1\DATA\KIRAN\KT316617.D

Sample Name: KST3166D1

Injection Date	: 10/12/2018 11:41:51 AM	Seq. Line :	1
Sample Name	: KST3166D1	Location :	Vial 1
Acq. Operator	: KIRAN	Inj :	1
Acq. Instrument	: Instrument 1	Inj Volume :	100 µl
Different Inj Volume from Sequence !		Actual Inj Volume :	10 µl
Sequence File	: C:\HPCHEM\1\SEQUENCE\DEF_LC.S		
Method	: C:\HPCHEM\1\METHODS\AN0010.M		
Last changed	: 10/12/2018 11:39:45 AM by KIRAN		

S6

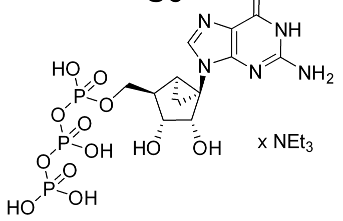

4a:(S)-mc-GTP; **MRS4589**

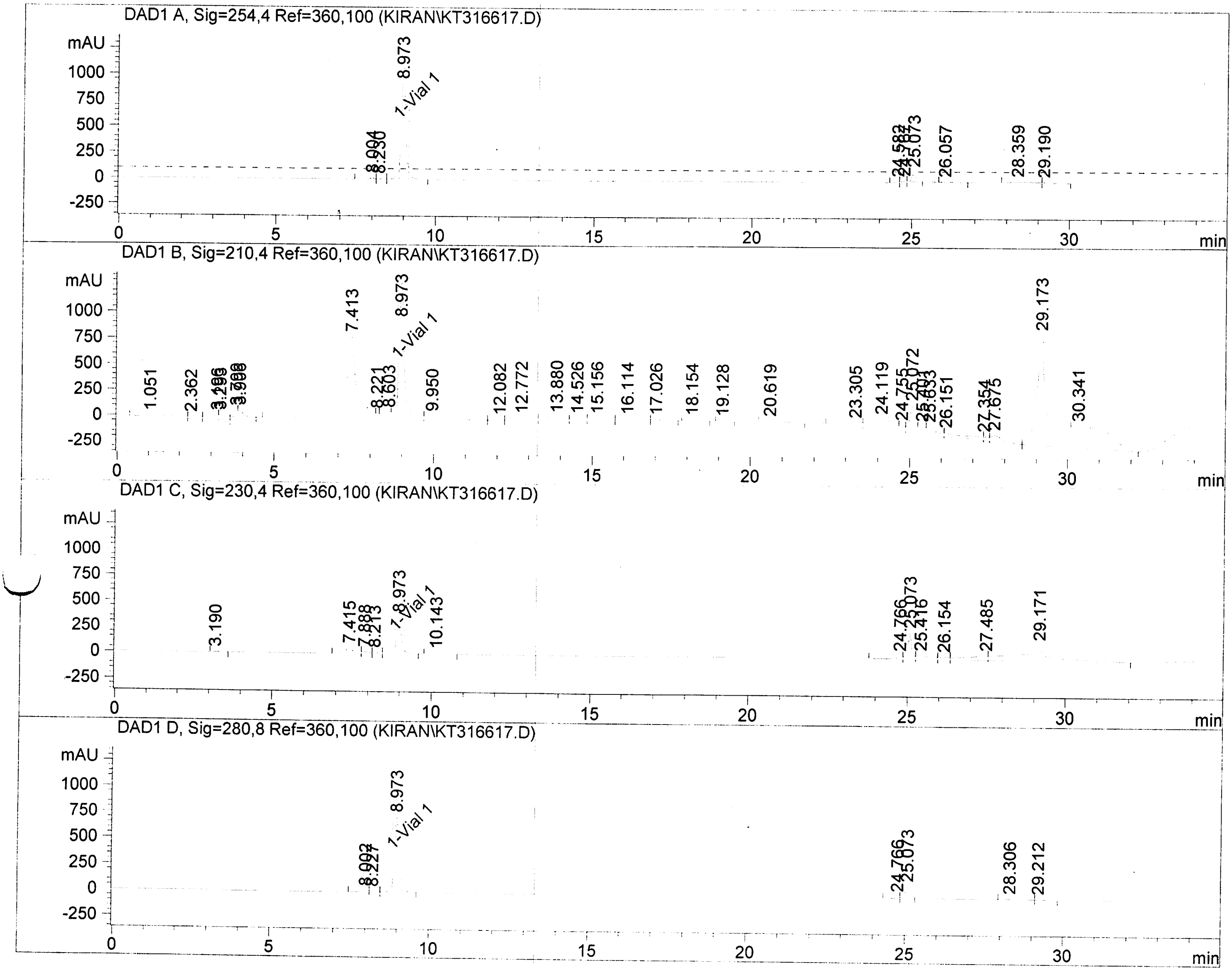

Fraction Information

Fraction collection peak controlled
Maximum peak duration 4 min.
Fractions are collected, when at least one peak detector detects a peak

Frac #	Well #	Location	Volume [µl]	BeginTime [min]	EndTime [min]	Reason	Mass
1	1	1-Vial 1	315.00	8.8535	9.1535	DAD1	

Injection Date : 10/12/2018 11:41:51 AM Seq. Line : 1
Sample Name : KST3166D1 Location : Vial 1
Acq. Operator : KIRAN Inj : 1
Acq. Instrument : Instrument 1 Inj Volume : 100 µl
Different Inj Volume from Sequence ! Actual Inj Volume : 10 µl
Sequence File : C:\HPCHEM\1\SEQUENCE\DEF_LC.S
Method : C:\HPCHEM\1\METHODS\AN0010.M
Last changed : 10/12/2018 11:39:45 AM by KIRAN

Area Percent Report

Sorted By : Signal
Multiplier : 1.0000
Dilution : 1.0000
Use Multiplier & Dilution Factor with ISTDs

Signal 1: DAD1 A, Sig=254,4 Ref=360,100

Peak #	RetTime [min]	Type	Width [min]	Area [mAU*s]	Height [mAU]	Area %
1	8.004	BV	0.1944	178.33264	12.06954	1.4326
2	8.230	VV	0.1765	78.80063	6.27470	0.6330
3	8.973	VB	0.1490	1.07524e4	1072.57935	86.3758
4	24.582	PV	0.1047	26.25638	3.89373	0.2109
5	24.767	VV	0.1384	108.12511	11.63821	0.8686
6	25.073	VB	0.1119	806.14734	96.02168	6.4759
7	26.057	PP	0.1155	16.06891	1.92265	0.1291
8	28.359	BV	0.6816	389.92679	7.08992	3.1323
9	29.190	VP	0.1805	92.33832	6.46844	0.7418

Totals : 1.24484e4 1217.95821

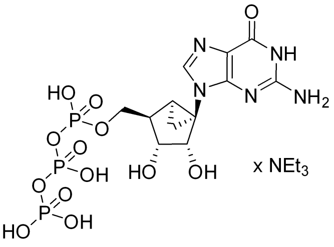

4a:(S)-mc-GTP; **MRS4589**

Results obtained with enhanced integrator!

Signal 2: DAD1 B, Sig=210,4 Ref=360,100

Peak #	RetTime [min]	Type	Width [min]	Area [mAU*s]	Height [mAU]	Area %
1	1.051	BV	1.2755	1053.19910	9.67898	0.9748
2	2.362	VV	0.3122	399.98389	16.19422	0.3702
3	3.196	VV	0.2157	678.86737	41.31947	0.6284
4	3.293	VP	0.1792	499.81091	43.79323	0.4626
5	3.799	VV	0.1275	703.60803	85.94725	0.6513
6	3.906	VB	0.1812	1120.33972	85.34208	1.0370
7	7.413	BV	0.3150	1.83728e4	785.15985	17.0056
8	8.221	VV	0.0980	369.49396	52.54774	0.3420
9	8.603	VV	0.2689	1302.48047	64.14443	1.2056
10	8.973	VB	0.1714	1.55210e4	1318.22083	14.3661
11	9.950	BP	0.8075	2035.59729	34.04876	1.8841
12	12.082	BV	0.2960	111.09353	5.22539	0.1028
13	12.772	VV	0.6710	1036.44153	21.03090	0.9593
14	13.880	VV	0.6705	1382.46875	28.57165	1.2796
15	14.526	VV	0.4392	641.40985	20.95223	0.5937
16	15.156	VV	0.5831	818.55542	19.13401	0.7576
17	16.114	VV	0.6761	606.97870	10.69572	0.5618
18	17.026	VB	0.3338	151.47769	5.97445	0.1402
19	18.154	BP	0.3066	94.69833	4.51742	0.0877
20	19.128	PP	0.1870	21.72026	1.77249	0.0201
21	20.619	BB	0.5100	323.05261	8.56620	0.2990
22	23.305	BV	0.5217	1485.85413	34.69229	1.3753
23	24.119	VV	0.7218	4939.60937	97.70393	4.5720
24	24.755	VV	0.1529	688.27863	59.54269	0.6371
25	25.072	VV	0.1344	2663.80298	258.02444	2.4656

S8

Injection Date : 10/12/2018 11:41:51 AM Seq. Line : 1
Sample Name : KST3166D1 Location : Vial 1
Acq. Operator : KIRAN Inj : 1
Acq. Instrument : Instrument 1 Inj Volume : 100 µl
Different Inj Volume from Sequence ! Actual Inj Volume : 10 µl
Sequence File : C:\HPCHEM\1\SEQUENCE\DEF_LC.S
Method : C:\HPCHEM\1\METHODS\AN0010.M
Last changed : 10/12/2018 11:39:45 AM by KIRAN

Peak #	RetTime [min]	Type	Width [min]	Area [mAU*s]	Height [mAU]	Area %
26	25.401	VV	0.2064	1085.79285	72.63398	1.0050
27	25.633	VV	0.2698	1513.61853	71.85312	1.4010
28	26.151	VV	0.7176	2061.90576	34.48144	1.9085
29	27.354	VV	0.1498	377.64221	33.44045	0.3495
30	27.675	VP	0.3395	1446.68762	58.30543	1.3390
31	29.173	VV	0.3102	2.73315e4	1091.30774	25.2977
32	30.341	VP	1.0418	1.71996e4	208.19081	15.9198

Totals : 1.08039e5 4683.01363

Results obtained with enhanced integrator!

Signal 3: DAD1 C, Sig=230,4 Ref=360,100

Peak #	RetTime [min]	Type	Width [min]	Area [mAU*s]	Height [mAU]	Area %
1	3.190	BP	0.2466	47.67048	2.49825	0.2235
2	7.415	BV	0.2809	1006.69507	48.34402	4.7208
3	7.888	VV	0.2049	242.66095	16.00676	1.1379
4	8.213	VV	0.2031	104.67621	6.82017	0.4909
5	8.973	VB	0.1519	3624.17065	352.50552	16.9951
6	10.143	PB	0.3970	92.21033	3.11796	0.4324
7	24.766	BV	0.2197	492.60123	28.49256	2.3100
8	25.073	VV	0.1159	2184.98828	250.13223	10.2463
9	25.416	VV	0.4564	1169.18787	33.73066	5.4828
10	26.154	VV	0.2780	527.96765	23.87681	2.4758
11	27.485	VV	0.6339	2147.03711	40.93493	10.0683
12	29.171	VB	0.7813	9684.87988	147.64188	45.4162

Totals : 2.13247e4 954.10175

Results obtained with enhanced integrator!

Signal 4: DAD1 D, Sig=280,8 Ref=360,100

Peak #	RetTime [min]	Type	Width [min]	Area [mAU*s]	Height [mAU]	Area %
1	8.002	BV	0.2149	142.85364	8.55327	1.7007
2	8.227	VV	0.1696	56.37685	4.58135	0.6712
3	8.973	VB	0.1481	7175.03223	720.93640	85.4184
4	24.766	PV	0.1413	85.92663	9.00814	1.0230
5	25.073	VB	0.1035	792.15314	103.26379	9.4305
6	28.306	BP	0.4608	111.32475	2.98734	1.3253
7	29.212	VB	0.2074	36.19797	2.18374	0.4309

Totals : 8399.86519 851.51404

Results obtained with enhanced integrator!

Summed Peaks Report

Signal 1: DAD1 A, Sig=254,4 Ref=360,100

S9

```
=====================================================================
Injection Date  : 10/12/2018 11:41:51 AM        Seq. Line :   1
Sample Name     : KST3166D1                      Location : Vial 1
Acq. Operator   : KIRAN                               Inj :   1
Acq. Instrument : Instrument 1                 Inj Volume : 100 µl
Different Inj Volume from Sequence !    Actual Inj Volume : 10 µl
Sequence File   : C:\HPCHEM\1\SEQUENCE\DEF_LC.S
Method          : C:\HPCHEM\1\METHODS\AN0010.M
Last changed    : 10/12/2018 11:39:45 AM by KIRAN
=====================================================================

        Signal 2: DAD1 B, Sig=210,4 Ref=360,100
        Signal 3: DAD1 C, Sig=230,4 Ref=360,100
        Signal 4: DAD1 D, Sig=280,8 Ref=360,100
=====================================================================
                      Final Summed Peaks Report
=====================================================================

        Signal 1: DAD1 A, Sig=254,4 Ref=360,100
        Signal 2: DAD1 B, Sig=210,4 Ref=360,100
        Signal 3: DAD1 C, Sig=230,4 Ref=360,100
        Signal 4: DAD1 D, Sig=280,8 Ref=360,100
                            *** End of Report ***
```

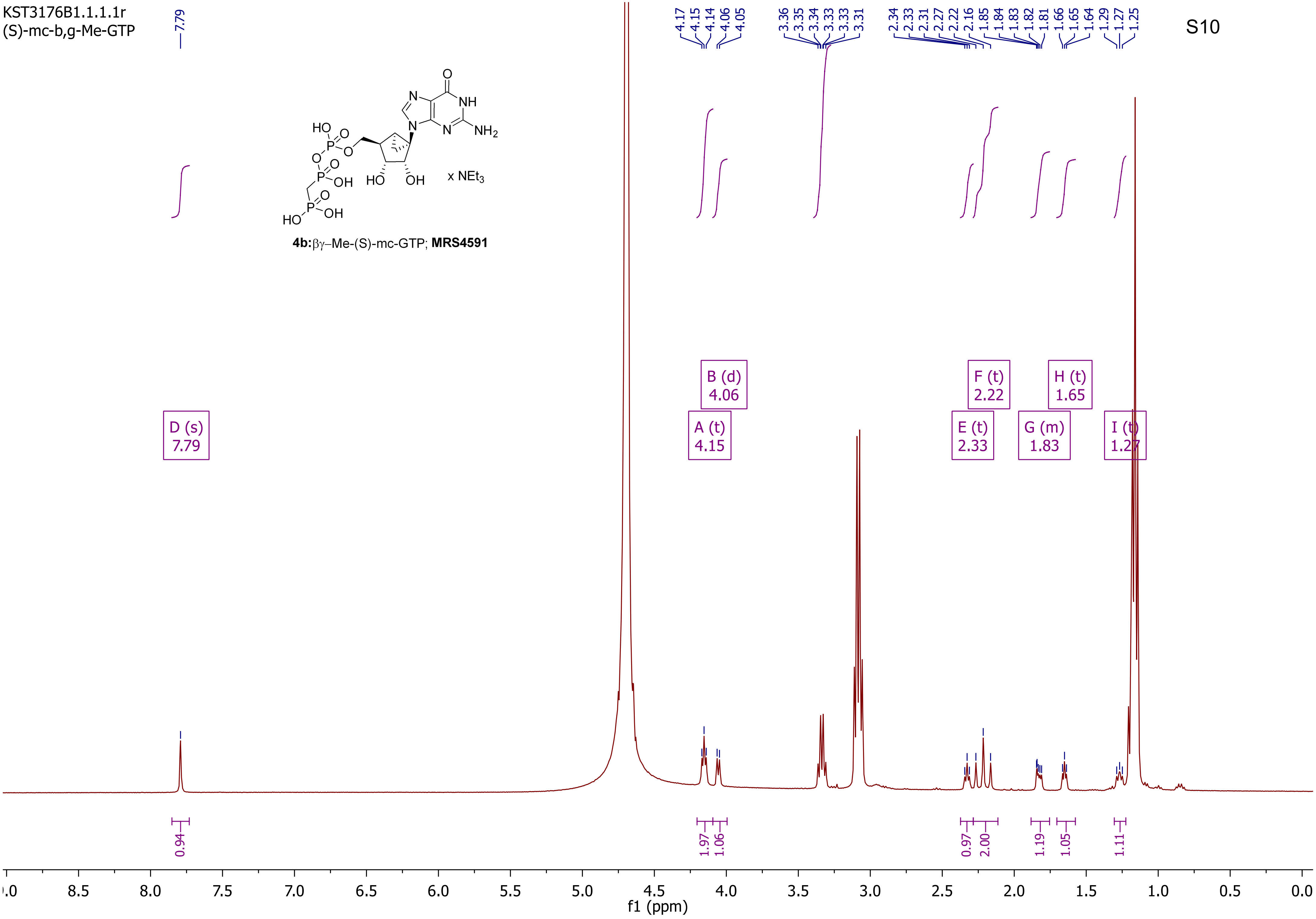

KST3176B1.1.1.1r
(S)-mc-b,g-Me-GTP
7.79
4.17 4.15 4.14 4.06 4.05
3.36 3.35 3.34 3.33 3.33 3.31
2.34 2.33 2.31 2.27 2.22 2.16 1.85 1.84 1.83 1.82 1.81 1.66 1.65 1.64 1.29 1.27 1.25
HO O P O O P O OH O P OH HO OH HO N N NH O N NH2
x NEt3
4b:βγ–Me-(S)-mc-GTP; MRS4591
D (s) 7.79
A (t) 4.15
B (d) 4.06
E (t) 2.33
F (t) 2.22
G (m) 1.83
H (t) 1.65
I (t) 1.27
0.94
1.97
1.06
0.97
2.00
1.19
1.05
1.11
8.5 8.0 7.5 7.0 6.5 6.0 5.5 5.0 4.5 4.0 3.5 3.0 2.5 2.0 1.5 1.0 0.5 0.0
f1 (ppm)

KST3176B1-p31.1.1.1r
(S)-mc-b,g-Me-GTP

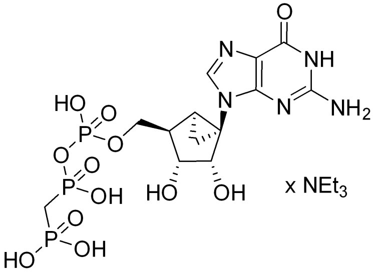

4b:βγ–Me-(S)-mc-GTP; **MRS4591**

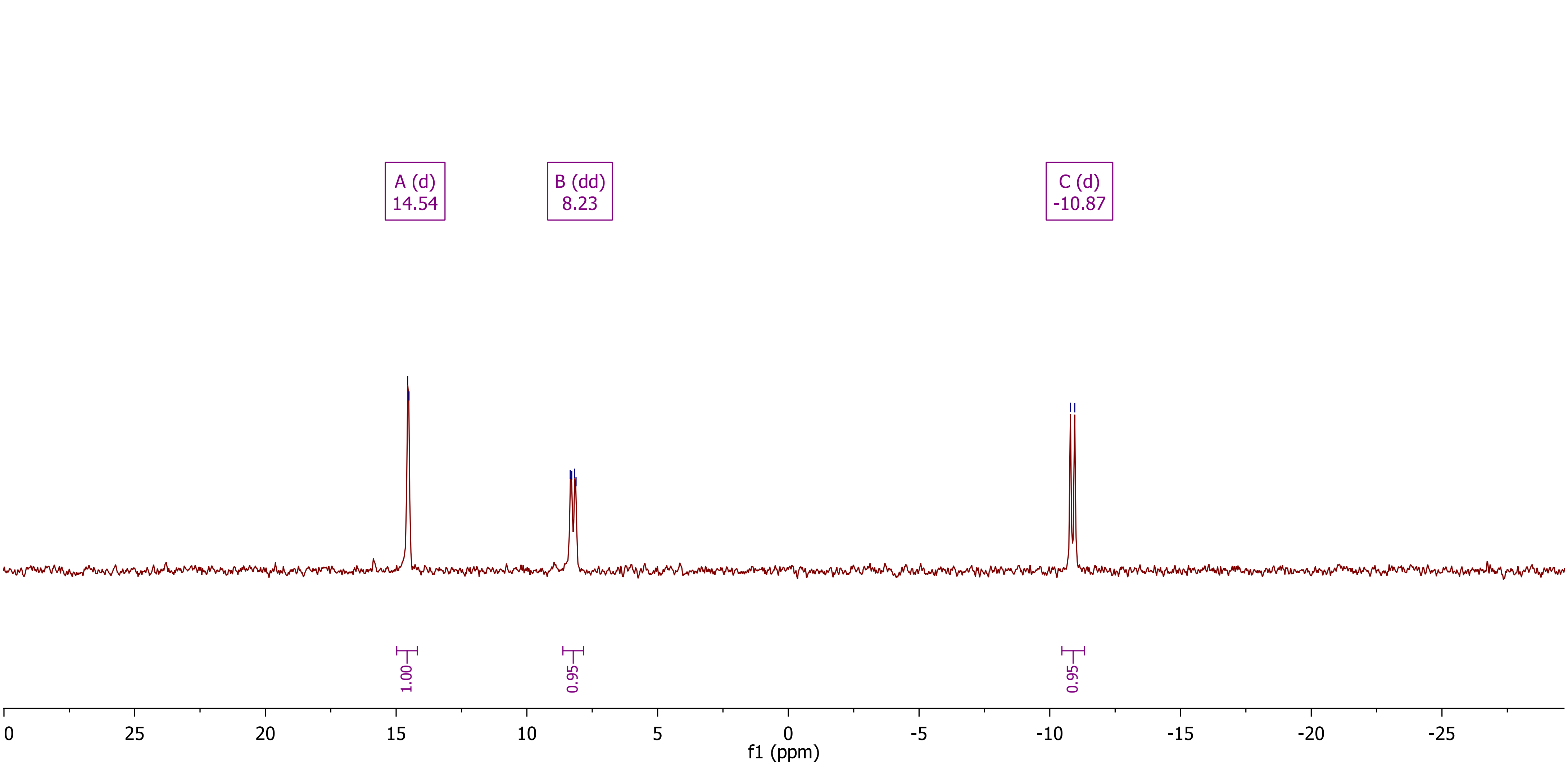

Elemental Composition Report

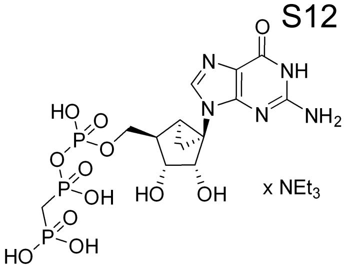

4b:βγ–Me-(S)-mc-GTP; **MRS4591**

Single Mass Analysis

Tolerance = 5.0 mDa / DBE: min = -1.5, max = 100.0
Element prediction: Off
Number of isotope peaks used for i-FIT = 3

Monoisotopic Mass, Even Electron Ions
91 formula(e) evaluated with 1 results within limits (up to 50 closest results for each mass)
Elements Used:
C: 0-150 H: 0-200 N: 5-5 O: 0-60 P: 3-3
KST-16NOV18-3-176-B 145 (2.470) AM2 (Ar,22000.0,0.00,0.00); ABS
TOF MS ES-

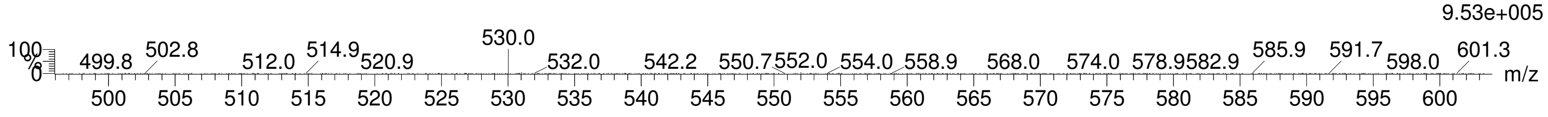

	Calc. Mass	mDa	PPM	DBE	i-FIT	Norm	Conf(%)	Formula
Minimum:				-1.5				
Maximum:		5.0	5.0	100.0				
Mass	Calc. Mass	mDa	PPM	DBE	i-FIT	Norm	Conf(%)	Formula
530.0250	530.0243	0.7	1.3	8.5	176.6	n/a	n/a	C13 H19 N5 O12 P3

S13 kc3176b1

```
Injection Date   : 11/21/2018 2:17:21 PM        Seq. Line :   2
Sample Name      : MRS4591                       Location : Vial 2
Acq. Operator    : KIRAN                              Inj :   1
Acq. Instrument  : Instrument 1                Inj Volume : 100 µl
Different Inj Volume from Sequence !   Actual Inj Volume : 10 µl
Sequence File    : C:\HPCHEM\1\SEQUENCE\DEF_LC.S
Method           : C:\HPCHEM\1\METHODS\AN0015.M
Last changed     : 11/21/2018 1:33:42 PM by KIRAN
```

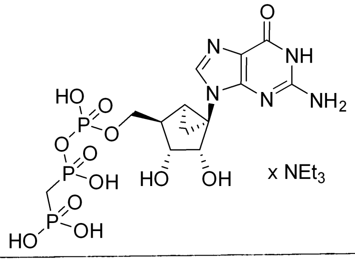

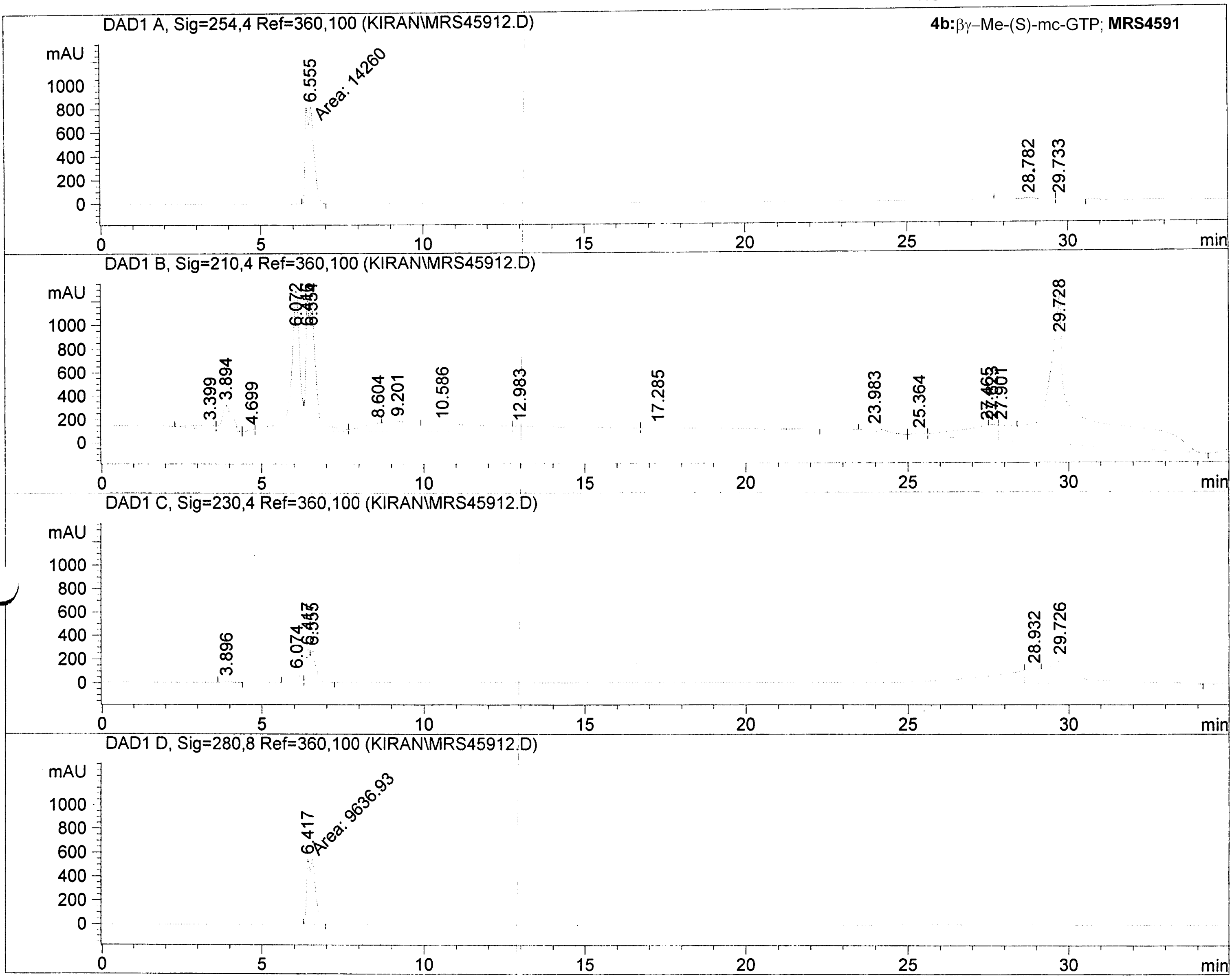

Fraction Information

Fraction collection off

No Fractions found.

S14

```
=====================================================================
Injection Date  : 11/21/2018 2:17:21 PM          Seq. Line :   2
Sample Name     : MRS4591                          Location : Vial 2
Acq. Operator   : KIRAN                                 Inj :   1
Acq. Instrument : Instrument 1                   Inj Volume : 100 µl
Different Inj Volume from Sequence !      Actual Inj Volume : 10 µl
Sequence File   : C:\HPCHEM\1\SEQUENCE\DEF_LC.S
Method          : C:\HPCHEM\1\METHODS\AN0015.M
Last changed    : 11/21/2018 1:33:42 PM by KIRAN
=====================================================================
```

Area Percent Report

Sorted By : Signal
Multiplier : 1.0000
Dilution : 1.0000
Use Multiplier & Dilution Factor with ISTDs

Signal 1: DAD1 A, Sig=254,4 Ref=360,100

Peak #	RetTime [min]	Type	Width [min]	Area [mAU*s]	Height [mAU]	Area %
1	6.555	MM	0.2904	1.42600e4	818.36975	94.4012
2	28.782	BV	0.9348	718.69122	9.65122	4.7577
3	29.733	VB	0.1986	127.04724	8.11655	0.8411

Totals : 1.51057e4 836.13752

Results obtained with enhanced integrator!

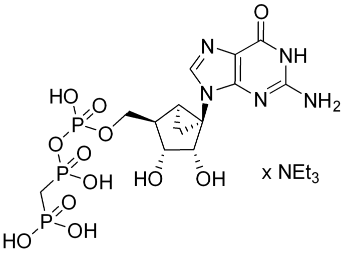

4b:βγ–Me-(S)-mc-GTP; **MRS4591**

Signal 2: DAD1 B, Sig=210,4 Ref=360,100

Peak #	RetTime [min]	Type	Width [min]	Area [mAU*s]	Height [mAU]	Area %
1	3.399	BV	0.5628	1602.12988	35.88866	0.9138
2	3.894	VP	0.2808	4383.15674	214.17645	2.5000
3	4.699	VV	0.2085	237.16504	17.46136	0.1353
4	6.072	VV	0.2642	2.19262e4	1194.29504	12.5058
5	6.416	VV	0.1057	8280.60059	1126.78357	4.7229
6	6.554	VB	0.1711	1.39519e4	1091.77954	7.9576
7	8.604	BV	0.6387	2982.06030	70.90079	1.7008
8	9.201	VV	0.8960	5212.57471	84.45751	2.9730
9	10.586	VV	1.7569	8577.17285	58.76177	4.8921
10	12.983	VV	2.0944	7242.05127	40.76911	4.1306
11	17.285	VB	2.0528	3887.98364	22.46917	2.2175
12	23.983	BP	0.6300	861.73468	22.04852	0.4915
13	25.364	VV	0.3450	561.61230	20.66940	0.3203
14	27.465	VV	0.7445	7544.31592	122.49615	4.3030
15	27.623	VV	0.2421	2364.59595	132.56717	1.3487
16	27.901	VV	0.4293	4564.24609	133.34140	2.6033
17	29.728	VP	0.7617	8.11487e4	1273.03931	46.2839

Totals : 1.75328e5 5661.90492

Results obtained with enhanced integrator!

Signal 3: DAD1 C, Sig=230,4 Ref=360,100

Peak #	RetTime [min]	Type	Width [min]	Area [mAU*s]	Height [mAU]	Area %
1	3.896	BP	0.2577	203.73988	11.12093	0.9449
2	6.074	BV	0.2143	1142.51746	76.46857	5.2988
3	6.417	VV	0.0983	1889.19141	274.59308	8.7618
4	6.555	VB	0.1482	2921.80396	270.08423	13.5509

S15

```
=====================================================================
Injection Date  : 11/21/2018 2:17:21 PM        Seq. Line :   2
Sample Name     : MRS4591                       Location : Vial 2
Acq. Operator   : KIRAN                              Inj :   1
Acq. Instrument : Instrument 1                Inj Volume : 100 µl
Different Inj Volume from Sequence !   Actual Inj Volume : 10 µl
Sequence File   : C:\HPCHEM\1\SEQUENCE\DEF_LC.S
Method          : C:\HPCHEM\1\METHODS\AN0015.M
Last changed    : 11/21/2018 1:33:42 PM by KIRAN
=====================================================================
```

Peak #	RetTime [min]	Type	Width [min]	Area [mAU*s]	Height [mAU]	Area %
5	28.932	BV	0.4953	3888.25562	124.19200	18.0331
6	29.726	VB	0.6947	1.15163e4	198.55765	53.4106

Totals : 2.15618e4 955.01646

Results obtained with enhanced integrator!

Signal 4: DAD1 D, Sig=280,8 Ref=360,100

Peak #	RetTime [min]	Type	Width [min]	Area [mAU*s]	Height [mAU]	Area %
1	6.417	MM	0.2911	9636.93262	551.71722	100.0000

Totals : 9636.93262 551.71722

Results obtained with enhanced integrator!

Summed Peaks Report

Signal 1: DAD1 A, Sig=254,4 Ref=360,100
Signal 2: DAD1 B, Sig=210,4 Ref=360,100
Signal 3: DAD1 C, Sig=230,4 Ref=360,100
Signal 4: DAD1 D, Sig=280,8 Ref=360,100

Final Summed Peaks Report

Signal 1: DAD1 A, Sig=254,4 Ref=360,100
Signal 2: DAD1 B, Sig=210,4 Ref=360,100
Signal 3: DAD1 C, Sig=230,4 Ref=360,100
Signal 4: DAD1 D, Sig=280,8 Ref=360,100

*** End of Report ***

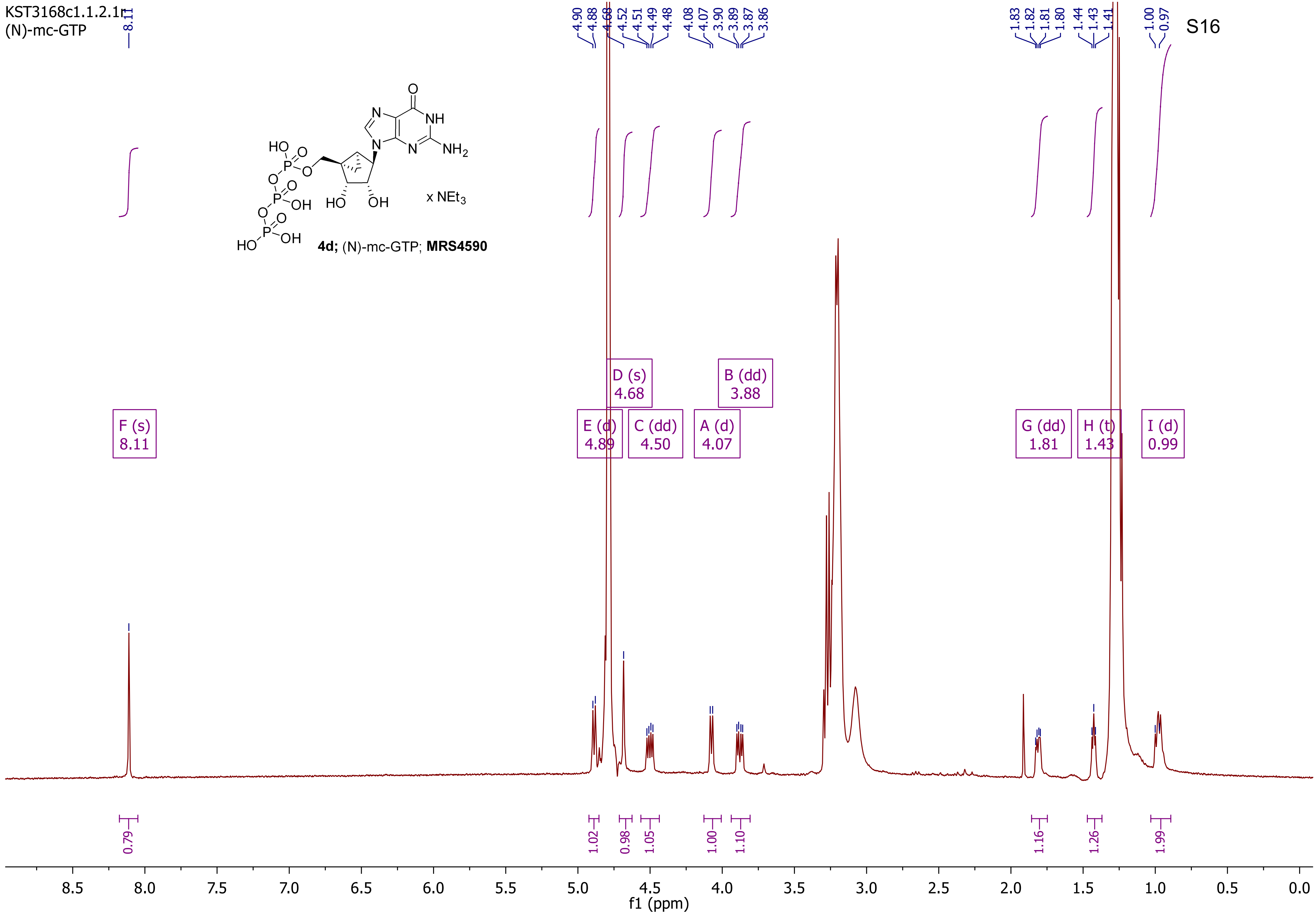

KST3168c1.1.2.1r
(N)-mc-GTP
8.11
4.90
4.88
4.68
4.52
4.51
4.49
4.48
4.08
4.07
3.90
3.89
3.87
3.86
1.83
1.82
1.81
1.80
1.44
1.43
1.41
1.00
0.97
x NEt3
4d; (N)-mc-GTP; MRS4590
F (s) 8.11
E (d) 4.89
D (s) 4.68
C (dd) 4.50
A (d) 4.07
B (dd) 3.88
G (dd) 1.81
H (t) 1.43
I (d) 0.99
0.79
1.02
0.98
1.05
1.00
1.10
1.16
1.26
1.99
8.5
8.0
7.5
7.0
6.5
6.0
5.5
5.0
4.5
4.0
3.5
3.0
2.5
2.0
1.5
1.0
0.5
0.0
f1 (ppm)

KST3168c1-p31.1.fid
(N)-mc-GTP

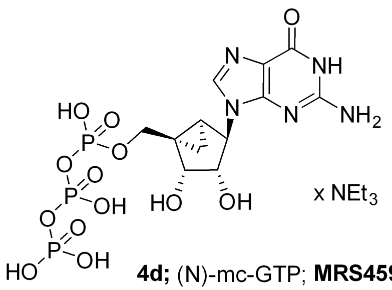

4d; (N)-mc-GTP; **MRS4590**

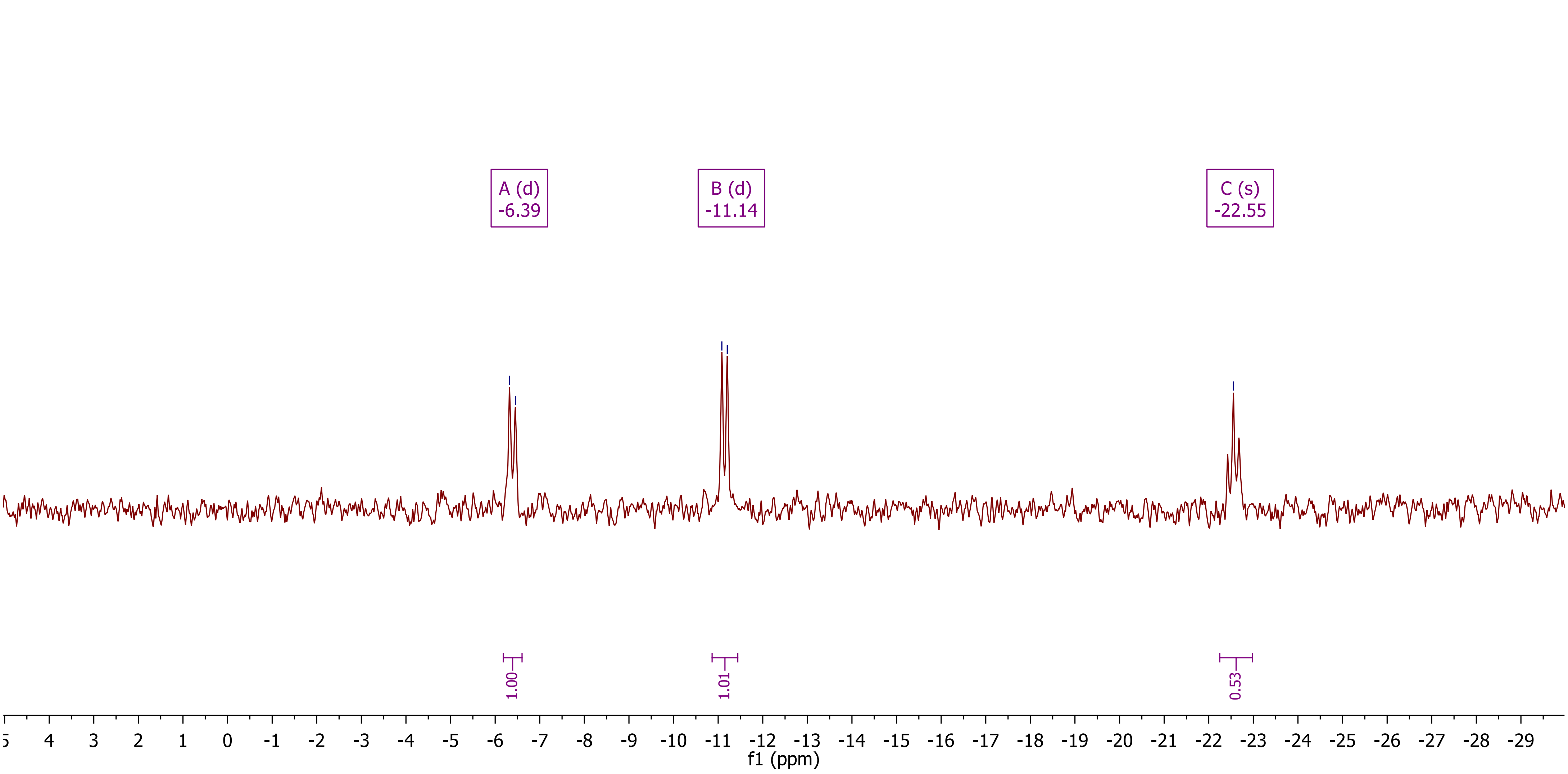

Elemental Composition Report

Single Mass Analysis
Tolerance = 10.0 mDa / DBE: min = -1.5, max = 100.0
Element prediction: Off
Number of isotope peaks used for i-FIT = 3

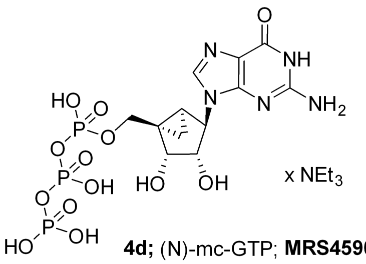

4d; (N)-mc-GTP; **MRS4590**

Monoisotopic Mass, Even Electron Ions
89 formula(e) evaluated with 5 results within limits (up to 50 closest results for each mass)
Elements Used:
C: 0-150 H: 0-200 N: 5-5 O: 0-60 P: 3-3
KST-24OCT18-3-168 199 (3.383) AM2 (Ar,22000.0,0.00,0.00); ABS; Cm (199-204x3.000)
TOF MS ES-

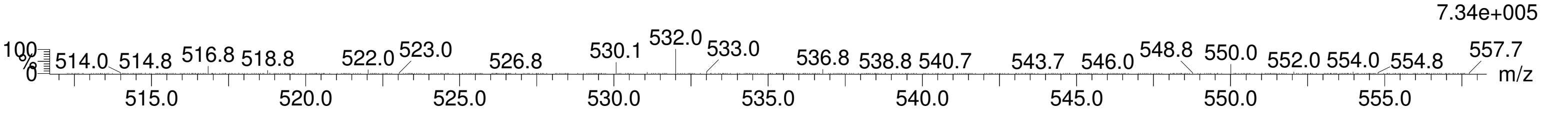

Minimum:				-1.5				
Maximum:		10.0	5.0	100.0				
Mass	Calc. Mass	mDa	PPM	DBE	i-FIT	Norm	Conf(%)	Formula
532.0034	532.0036	-0.2	-0.4	8.5	280.7	1.296	27.36	C12 H17 N5 O13 P3
	532.0071	-3.7	-7.0	30.5	285.0	5.524	0.40	C30 H9 N5 P3
	531.9977	5.7	10.7	17.5	282.9	3.496	3.03	C19 H13 N5 O8 P3
	532.0094	-6.0	-11.3	-0.5	279.8	0.376	68.64	C5 H21 N5 O18 P3
	532.0130	-9.6	-18.0	21.5	284.6	5.168	0.57	C23 H13 N5 O5 P3

```
=====================================================================
Injection Date   : 10/29/2018 2:03:02 PM           Seq. Line :   1
Sample Name      : KST3168C1                        Location : Vial 1
Acq. Operator    : KIRAN                                 Inj :   1
Acq. Instrument  : Instrument 1                   Inj Volume : 100 µl
Different Inj Volume from Sequence !       Actual Inj Volume : 10 µl
Sequence File    : C:\HPCHEM\1\SEQUENCE\DEF_LC.S
Method           : C:\HPCHEM\1\METHODS\AN0010.M
Last changed     : 10/29/2018 1:58:46 PM by KIRAN
=====================================================================
```

S19

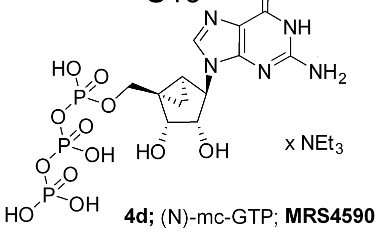

4d; (N)-mc-GTP; **MRS4590**

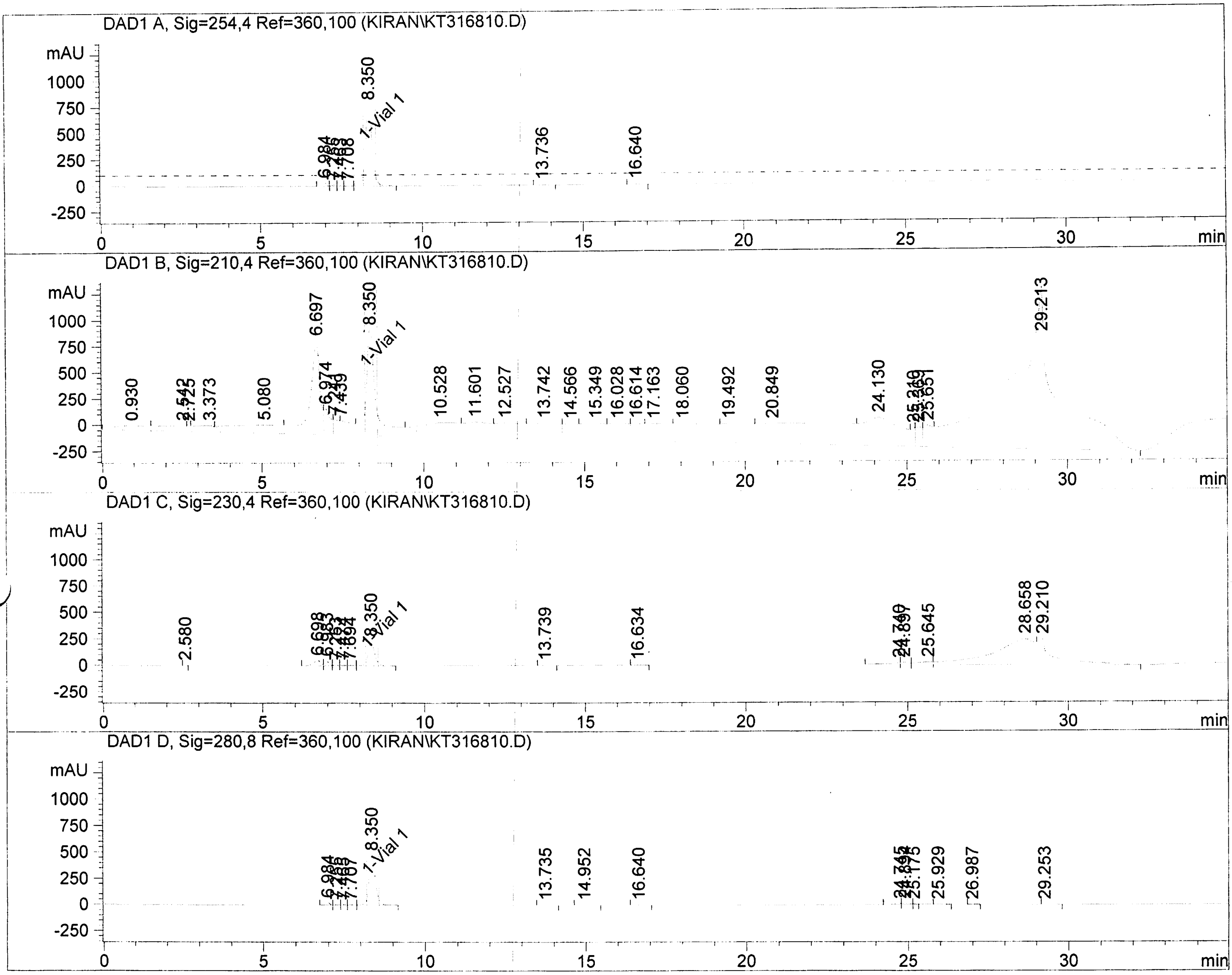


```
=====================================================================
                       Fraction Information
=====================================================================
Fraction collection peak controlled
Maximum peak duration 4 min.
Fractions are collected, when at least one peak detector detects a peak
=====================================================================
```

Frac #	Well #	Location	Volume [µl]	BeginTime [min]	EndTime [min]	Reason	Mass
1	1	1-Vial 1	388.54	8.1935	8.5671	DAD1	

S20

```
=====================================================================
Injection Date  : 10/29/2018 2:03:02 PM        Seq. Line :   1
Sample Name     : KST3168C1                     Location : Vial 1
Acq. Operator   : KIRAN                              Inj :   1
Acq. Instrument : Instrument 1                Inj Volume : 100 µl
Different Inj Volume from Sequence !   Actual Inj Volume : 10 µl
Sequence File   : C:\HPCHEM\1\SEQUENCE\DEF_LC.S
Method          : C:\HPCHEM\1\METHODS\AN0010.M
Last changed    : 10/29/2018 1:58:46 PM by KIRAN
=====================================================================
```

```
=====================================================================
                         Area Percent Report
=====================================================================

Sorted By             :      Signal
Multiplier            :      1.0000
Dilution              :      1.0000
Use Multiplier & Dilution Factor with ISTDs
```

Signal 1: DAD1 A, Sig=254,4 Ref=360,100

Peak #	RetTime [min]	Type	Width [min]	Area [mAU*s]	Height [mAU]	Area %
1	6.984	BV	0.0990	225.05707	32.41987	2.0082
2	7.255	VV	0.1245	93.36584	10.84363	0.8331
3	7.463	VV	0.1357	64.37907	6.60344	0.5745
4	7.708	VV	0.1748	83.75539	6.74958	0.7474
5	8.350	VB	0.2049	1.06213e4	770.09058	94.7748
6	13.736	PB	0.2371	58.96265	3.79847	0.5261
7	16.640	PB	0.2193	60.06382	4.29052	0.5360
Totals :				1.12069e4	834.79609	

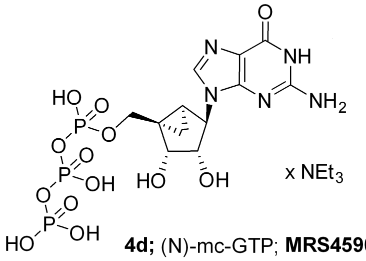

4d; (N)-mc-GTP; **MRS4590**

Results obtained with enhanced integrator!

Signal 2: DAD1 B, Sig=210,4 Ref=360,100

Peak #	RetTime [min]	Type	Width [min]	Area [mAU*s]	Height [mAU]	Area %
1	0.930	BV	0.8822	3061.28247	42.10805	0.7814
2	2.542	VV	0.7255	3275.00928	54.46832	0.8360
3	2.725	VV	0.1065	381.19550	51.38649	0.0973
4	3.373	VV	0.5029	2517.98315	62.11631	0.6427
5	5.080	VV	1.3873	9218.00684	78.30944	2.3530
6	6.697	VV	0.3107	1.95432e4	902.40100	4.9886
7	6.974	VV	0.1769	3331.91064	244.66580	0.8505
8	7.241	VV	0.1637	1821.59058	147.87582	0.4650
9	7.439	VV	0.3341	3580.31958	135.56551	0.9139
10	8.350	VB	0.2960	2.22037e4	1044.37256	5.6678
11	10.528	BV	1.1833	1.26388e4	137.64383	3.2262
12	11.601	VV	0.8090	8797.97461	151.17723	2.2458
13	12.527	VV	0.7942	8812.46387	148.09711	2.2495
14	13.742	VV	0.8146	1.00259e4	155.00137	2.5592
15	14.566	VV	0.4055	4754.67822	154.31708	1.2137
16	15.349	VV	0.6718	8336.83203	161.07726	2.1281
17	16.028	VV	0.5963	7266.52441	164.83006	1.8549
18	16.614	VV	0.3543	4373.76709	168.80350	1.1165
19	17.163	VV	0.6815	8822.52246	168.87540	2.2521
20	18.060	VV	1.0834	1.54463e4	173.71725	3.9428
21	19.492	VV	0.8060	1.18171e4	182.35114	3.0165
22	20.849	VV	2.3115	3.71373e4	192.43486	9.4797
23	24.130	VV	1.0699	2.14320e4	268.88361	5.4708
24	25.210	VV	0.1255	1564.47534	176.34169	0.3994
25	25.369	VV	0.1905	2596.48975	184.18478	0.6628
26	25.651	VV	0.2571	4016.47827	204.51772	1.0253
27	29.213	VP	1.1856	1.54980e5	1548.50171	39.5606

S21

```
=====================================================================
Injection Date  : 10/29/2018 2:03:02 PM          Seq. Line :   1
Sample Name     : KST3168C1                       Location : Vial 1
Acq. Operator   : KIRAN                                Inj :   1
Acq. Instrument : Instrument 1                  Inj Volume : 100 µl
Different Inj Volume from Sequence !     Actual Inj Volume : 10 µl
Sequence File   : C:\HPCHEM\1\SEQUENCE\DEF_LC.S
Method          : C:\HPCHEM\1\METHODS\AN0010.M
Last changed    : 10/29/2018 1:58:46 PM by KIRAN
=====================================================================
```

Peak #	RetTime [min]	Type	Width [min]	Area [mAU*s]	Height [mAU]	Area %
Totals :				3.91754e5	7104.02488	

Results obtained with enhanced integrator!

Signal 3: DAD1 C, Sig=230,4 Ref=360,100

Peak #	RetTime [min]	Type	Width [min]	Area [mAU*s]	Height [mAU]	Area %
1	2.580	BP	0.0835	11.37449	2.15732	0.0315
2	6.698	BV	0.2350	795.41589	51.26986	2.2015
3	6.983	VV	0.1135	351.72830	42.96552	0.9735
4	7.253	VV	0.1499	73.25932	6.58177	0.2028
5	7.474	VV	0.1716	74.30257	5.79390	0.2056
6	7.694	VV	0.1886	72.17373	5.17864	0.1998
7	8.350	VB	0.2082	3208.79492	227.95531	8.8809
8	13.739	BB	0.2541	17.96131	1.04726	0.0497
9	16.634	PP	0.1964	12.73746	1.02941	0.0353
10	24.740	BV	0.3141	369.98257	14.48000	1.0240
11	24.897	VV	0.1957	317.99911	22.12216	0.8801
12	25.645	VV	0.4417	823.62286	23.80437	2.2795
13	28.658	VV	1.1493	2.14270e4	242.86955	59.3033
14	29.210	VP	0.4293	8574.88184	242.06310	23.7326
Totals :				3.61313e4	889.31816	

Results obtained with enhanced integrator!

Signal 4: DAD1 D, Sig=280,8 Ref=360,100

Peak #	RetTime [min]	Type	Width [min]	Area [mAU*s]	Height [mAU]	Area %
1	6.984	BV	0.0996	188.88846	27.00465	2.5807
2	7.255	VV	0.1266	61.18793	6.96003	0.8360
3	7.465	VV	0.1395	41.45719	4.11762	0.5664
4	7.707	VV	0.1720	53.11303	4.30424	0.7256
5	8.350	VB	0.2047	6514.38916	472.91422	89.0017
6	13.735	PB	0.2318	36.13347	2.39781	0.4937
7	14.952	BB	0.3620	41.28629	1.55376	0.5641
8	16.640	PB	0.2229	38.21883	2.70478	0.5222
9	24.745	PV	0.1716	117.36393	8.80149	1.6035
10	24.894	VV	0.1789	151.24405	11.69575	2.0663
11	25.175	VB	0.1093	15.69675	1.92091	0.2145
12	25.929	PP	0.1561	13.72323	1.29062	0.1875
13	26.987	PP	0.1280	10.04806	1.19666	0.1373
14	29.253	PB	0.2193	36.64611	2.08257	0.5007
Totals :				7319.39648	548.94510	

Results obtained with enhanced integrator!

```
=====================================================================
                       Summed Peaks Report
=====================================================================
```

Signal 1: DAD1 A, Sig=254,4 Ref=360,100

S22

```
=====================================================================
Injection Date  : 10/29/2018 2:03:02 PM          Seq. Line :   1
Sample Name     : KST3168C1                       Location : Vial 1
Acq. Operator   : KIRAN                                Inj :   1
Acq. Instrument : Instrument 1                  Inj Volume : 100 µl
Different Inj Volume from Sequence !     Actual Inj Volume : 10 µl
Sequence File   : C:\HPCHEM\1\SEQUENCE\DEF_LC.S
Method          : C:\HPCHEM\1\METHODS\AN0010.M
Last changed    : 10/29/2018 1:58:46 PM by KIRAN
=====================================================================

        Signal 2: DAD1 B, Sig=210,4 Ref=360,100
        Signal 3: DAD1 C, Sig=230,4 Ref=360,100
        Signal 4: DAD1 D, Sig=280,8 Ref=360,100
=====================================================================
                      Final Summed Peaks Report
=====================================================================

        Signal 1: DAD1 A, Sig=254,4 Ref=360,100
        Signal 2: DAD1 B, Sig=210,4 Ref=360,100
        Signal 3: DAD1 C, Sig=230,4 Ref=360,100
        Signal 4: DAD1 D, Sig=280,8 Ref=360,100
                          *** End of Report ***
```

KST3152 1 1 /opt/topspin kiran

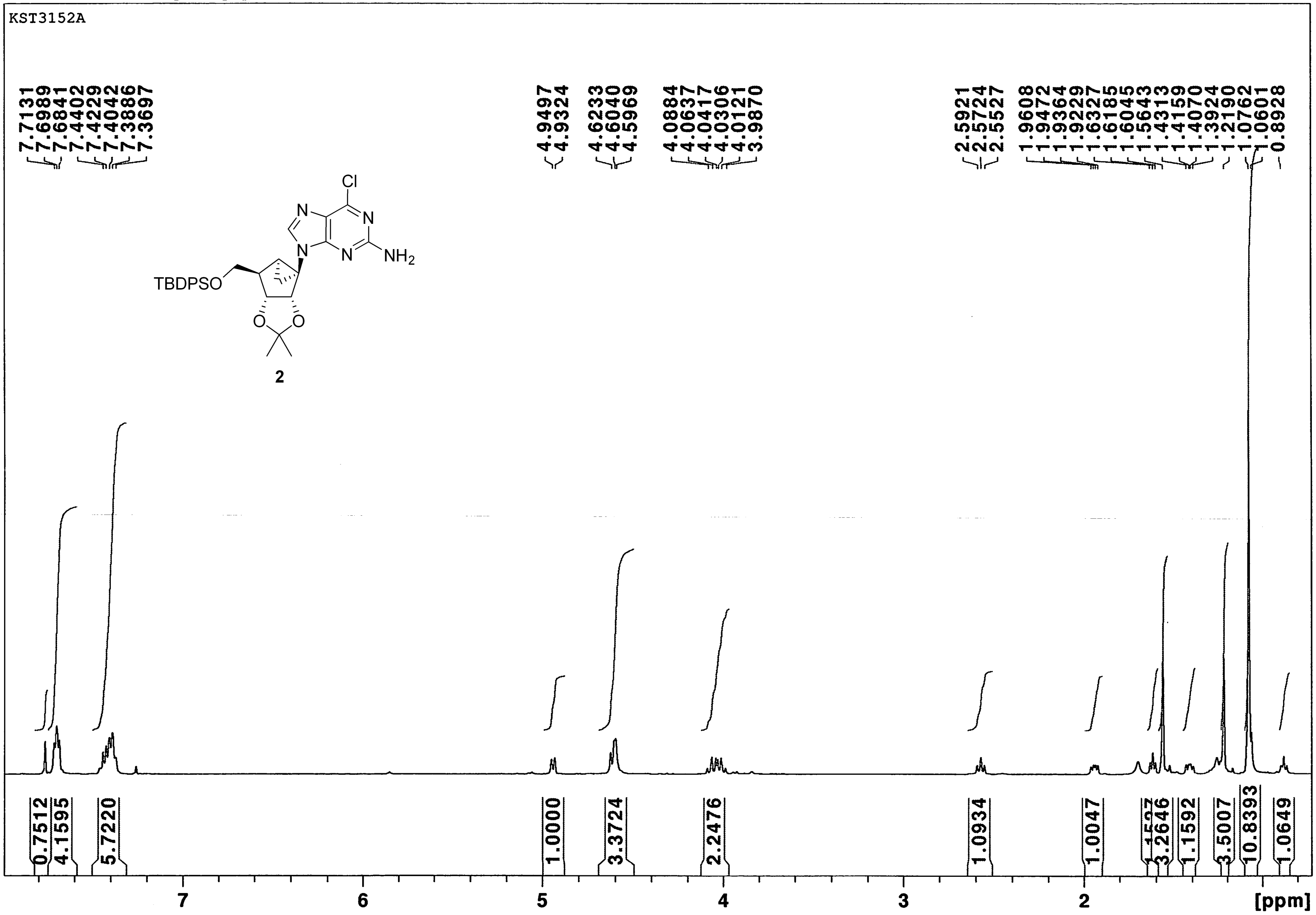

Elemental Composition Report

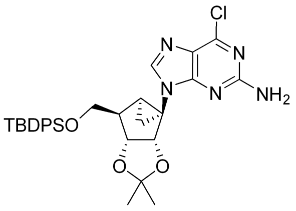

2

Single Mass Analysis

Tolerance = 5.0 PPM / DBE: min = -1.5, max = 100.0
Element prediction: Off
Number of isotope peaks used for i-FIT = 3

Monoisotopic Mass, Even Electron Ions
120 formula(e) evaluated with 1 results within limits (up to 50 closest results for each mass)
Elements Used:
C: 0-100 H: 0-200 N: 5-5 O: 0-40 28Si: 1-1 35Cl: 1-1
KST-16AUG18-5-152-A 224 (3.806) AM2 (Ar,42000.0,0.00,0.00); ABS
TOF MS ES+

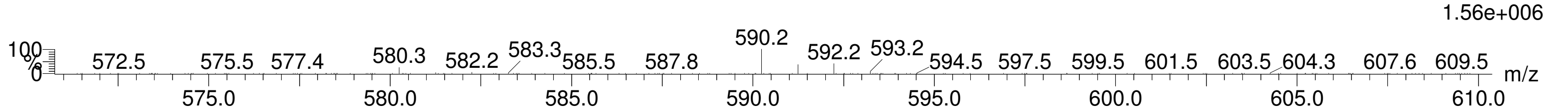

Minimum:				-1.5				
Maximum:		5.0	5.0	100.0				
Mass	Calc. Mass	mDa	PPM	DBE	i-FIT	Norm	Conf(%)	Formula
590.2353	590.2354	-0.1	-0.2	16.5	106.9	n/a	n/a	C31 H37 N5 O3 28Si 35Cl

KST3153.1.fid
(S)-mc-guanosine

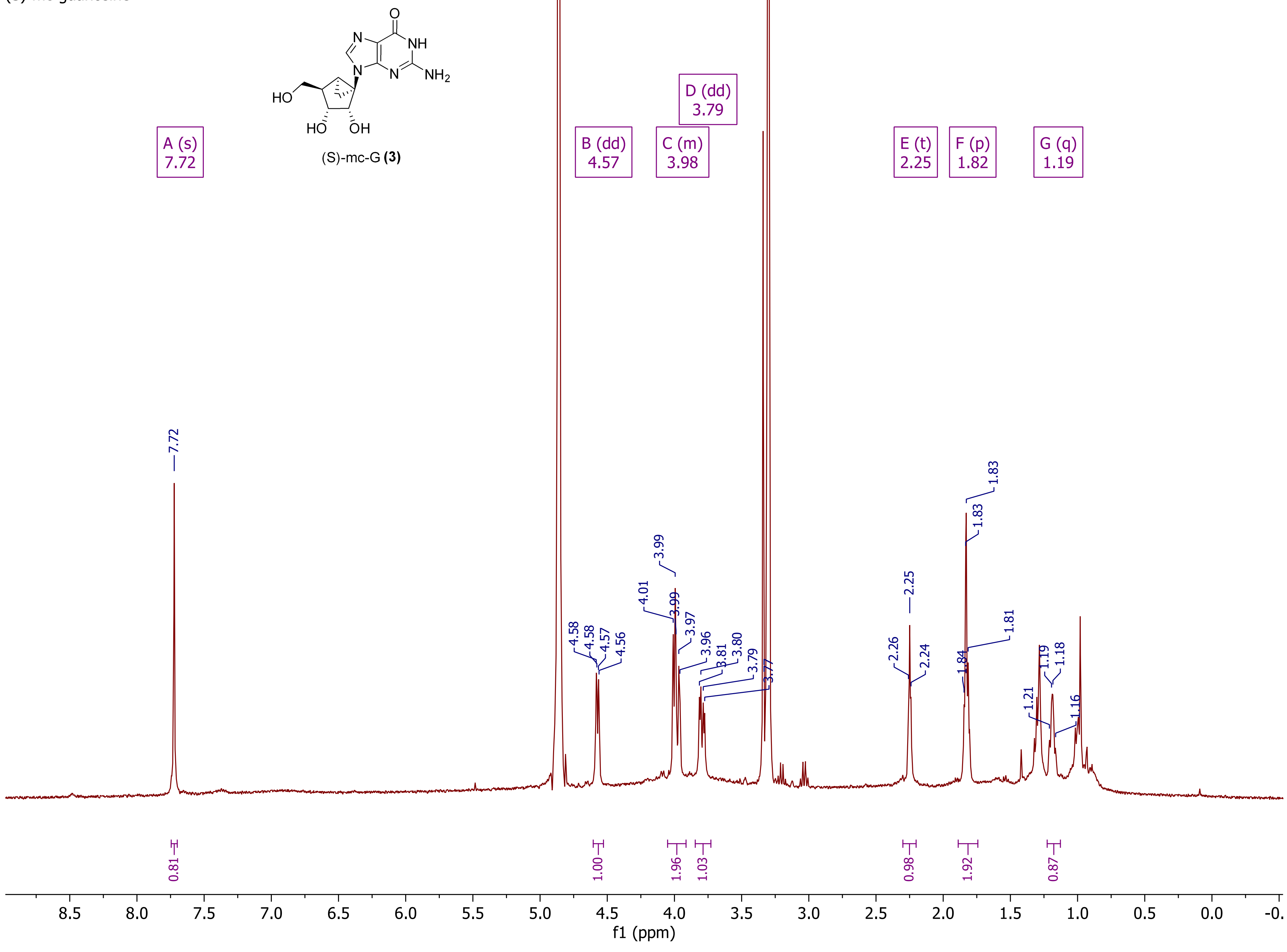

Elemental Composition Report

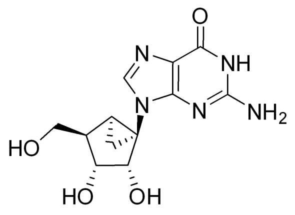

(S)-mc-G **(3)**

Single Mass Analysis

Tolerance = 5.0 PPM / DBE: min = -1.5, max = 100.0
Element prediction: Off
Number of isotope peaks used for i-FIT = 3

Monoisotopic Mass, Even Electron Ions
40 formula(e) evaluated with 1 results within limits (up to 50 closest results for each mass)
Elements Used:
C: 0-100 H: 0-200 N: 5-5 O: 0-40
KST-28AUG18-3-153 136 (2.317) AM2 (Ar,42000.0,0.00,0.00); ABS
TOF MS ES+

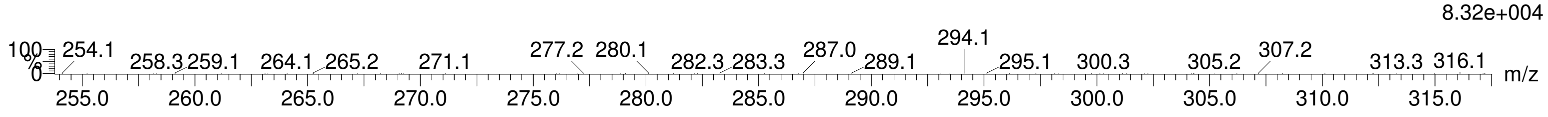

Minimum:				-1.5				
Maximum:		5.0	5.0	100.0				
Mass	Calc. Mass	mDa	PPM	DBE	i-FIT	Norm	Conf(%)	Formula
294.1201	294.1202	-0.1	-0.3	7.5	31.3	n/a	n/a	C12 H16 N5 O4

KST3170.1.fid
(S)-mc-1,2-isopropylidene-G

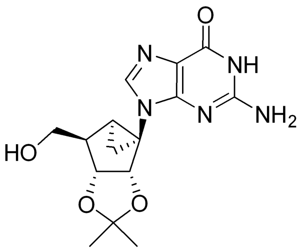

5

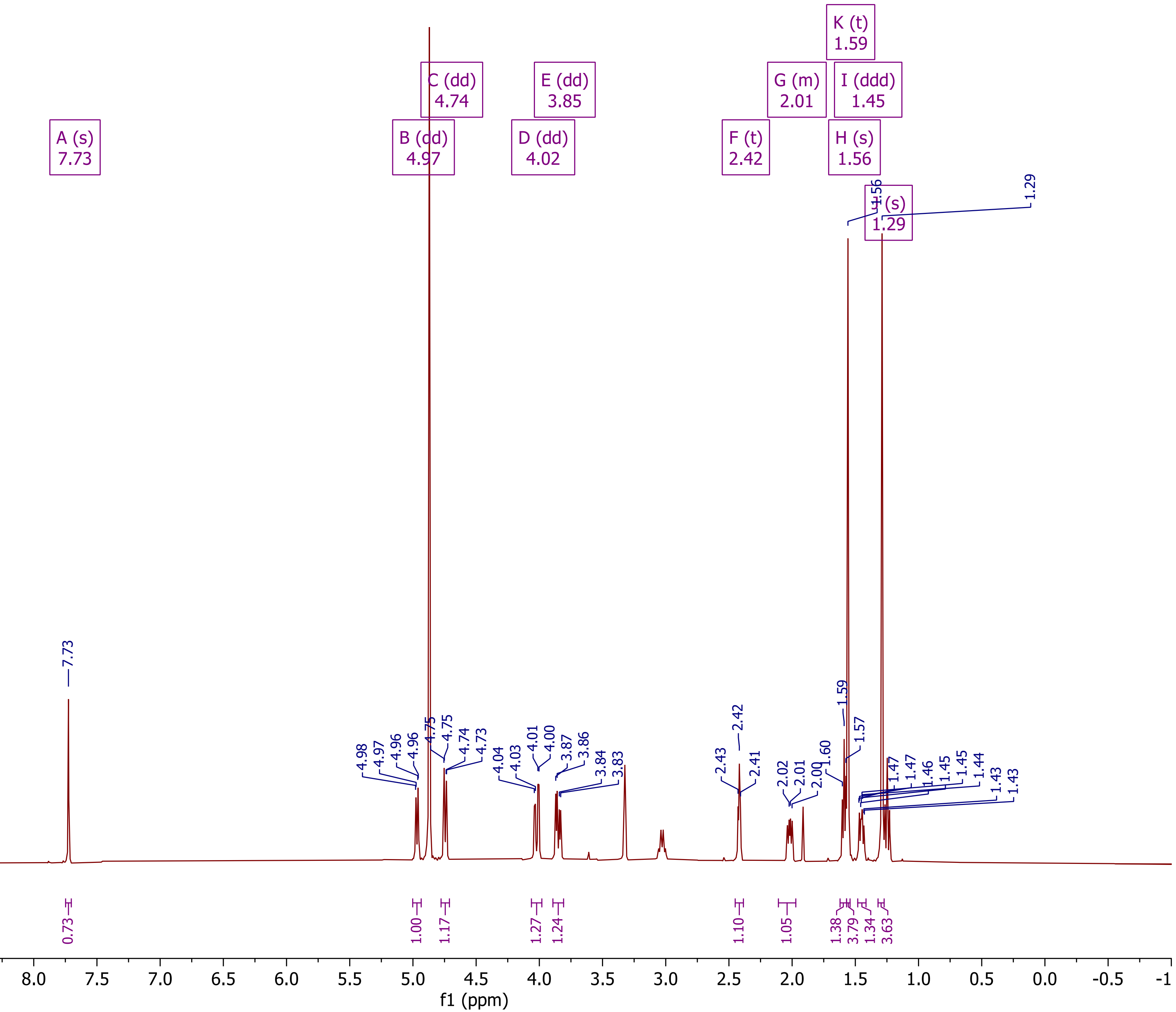

Elemental Composition Report

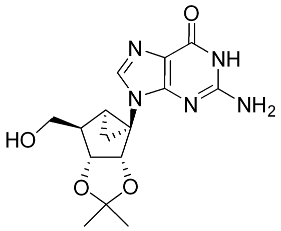

5

Single Mass Analysis

Tolerance = 5.0 mDa / DBE: min = -1.5, max = 100.0
Element prediction: Off
Number of isotope peaks used for i-FIT = 3

Monoisotopic Mass, Even Electron Ions
51 formula(e) evaluated with 1 results within limits (up to 50 closest results for each mass)
Elements Used:
C: 0-100 H: 0-250 N: 5-5 O: 0-60
KST-08JAN20-3-170 252 (4.280) AM2 (Ar,25000.0,0.00,0.00); ABS
TOF MS ES+

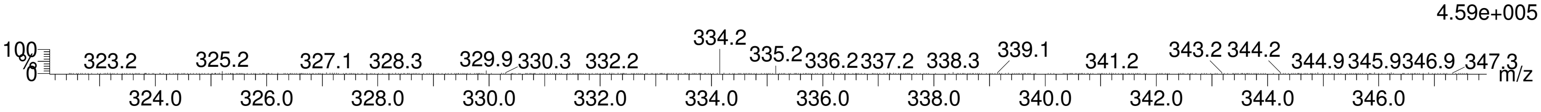

Minimum:				-1.5				
Maximum:		5.0	5.0	100.0				
Mass	Calc. Mass	mDa	PPM	DBE	i-FIT	Norm	Conf(%)	Formula
334.1519	334.1515	0.4	1.2	8.5	522.7	n/a	n/a	C15 H20 N5 O4

KST3172.1.fid

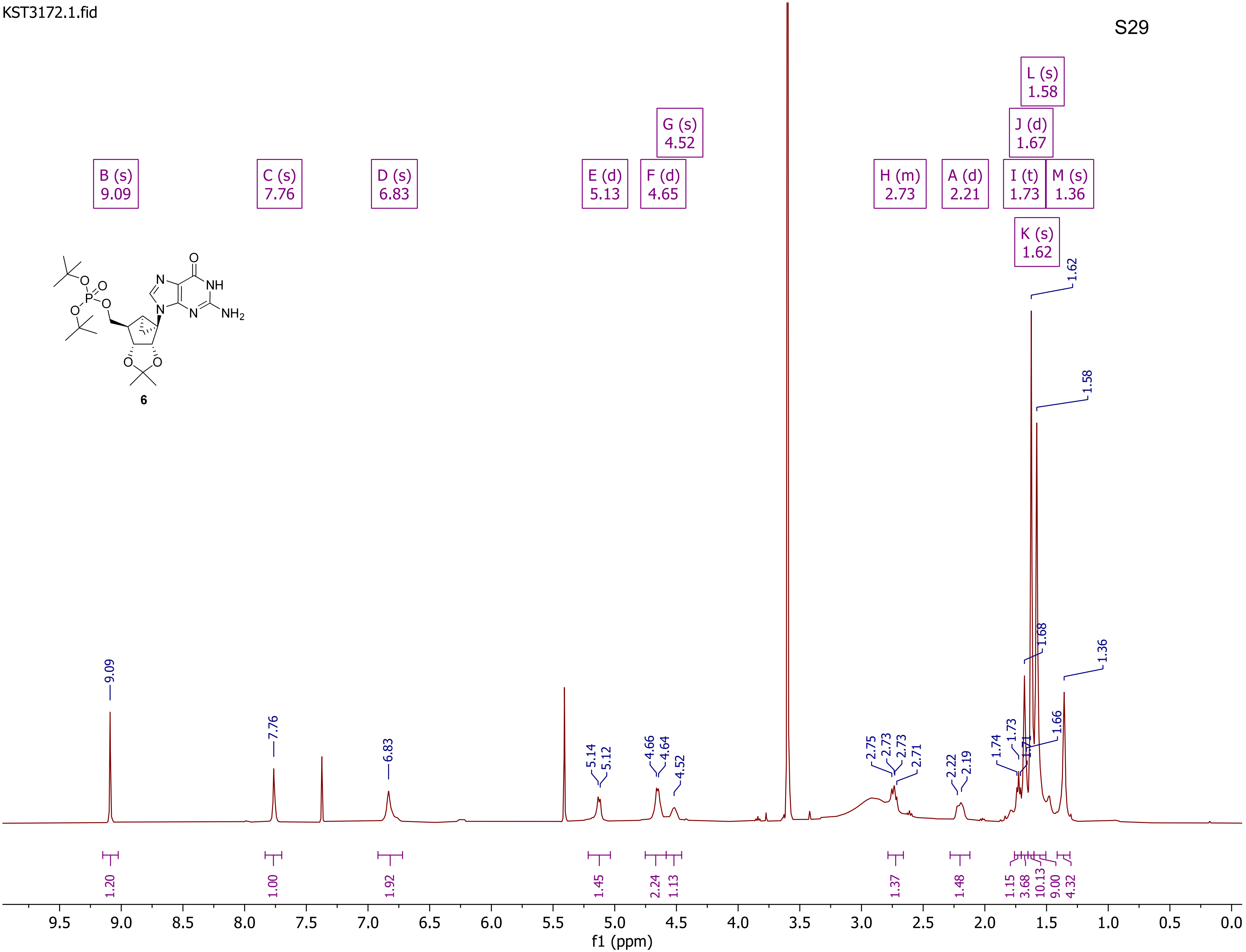

KST3172-p31.1.fid

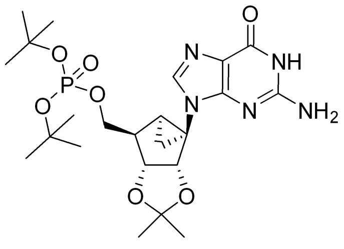

6

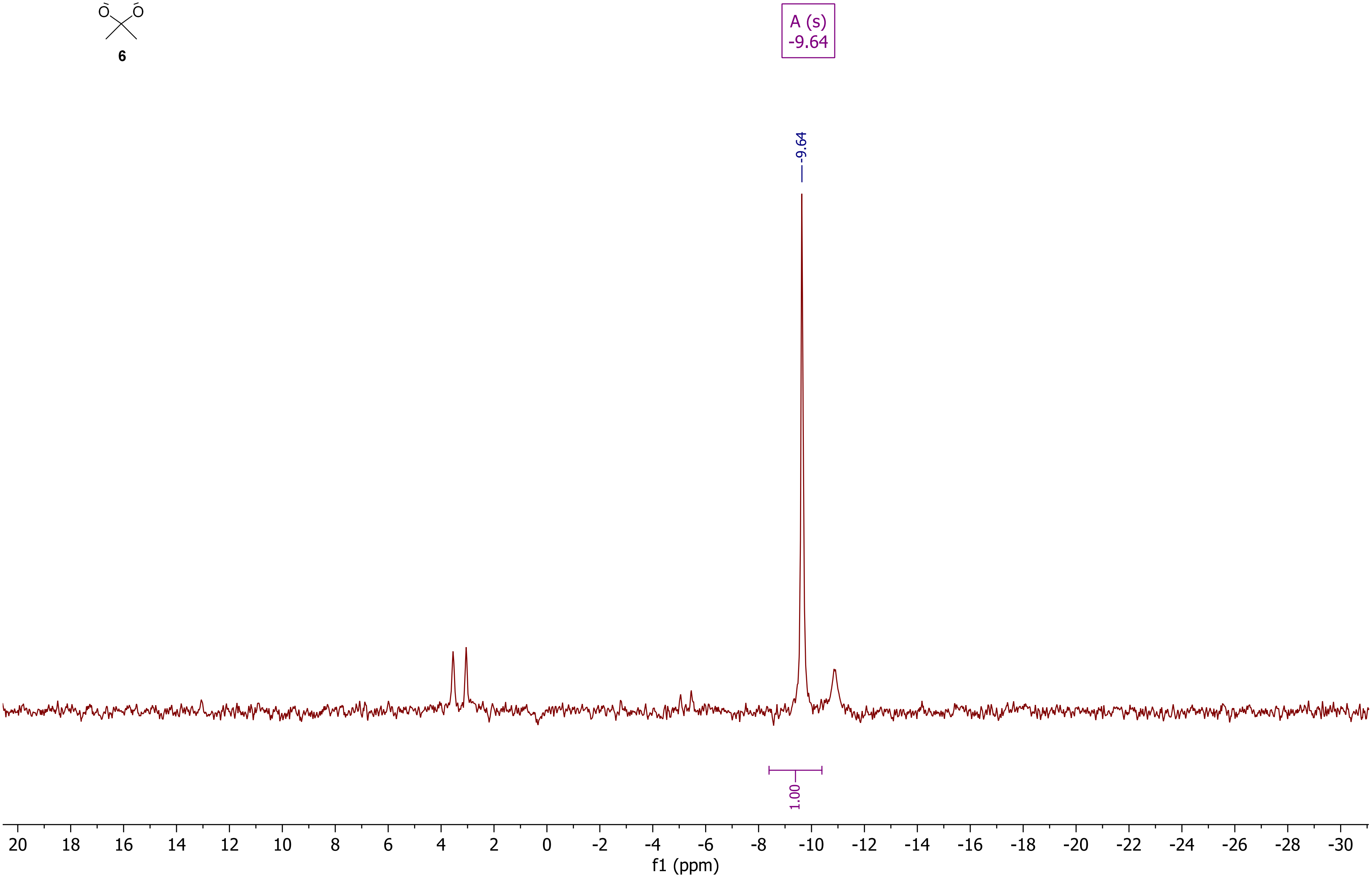

KST3174.1.fid

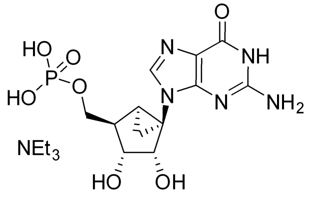

(S)-mc-GMP **(7)**

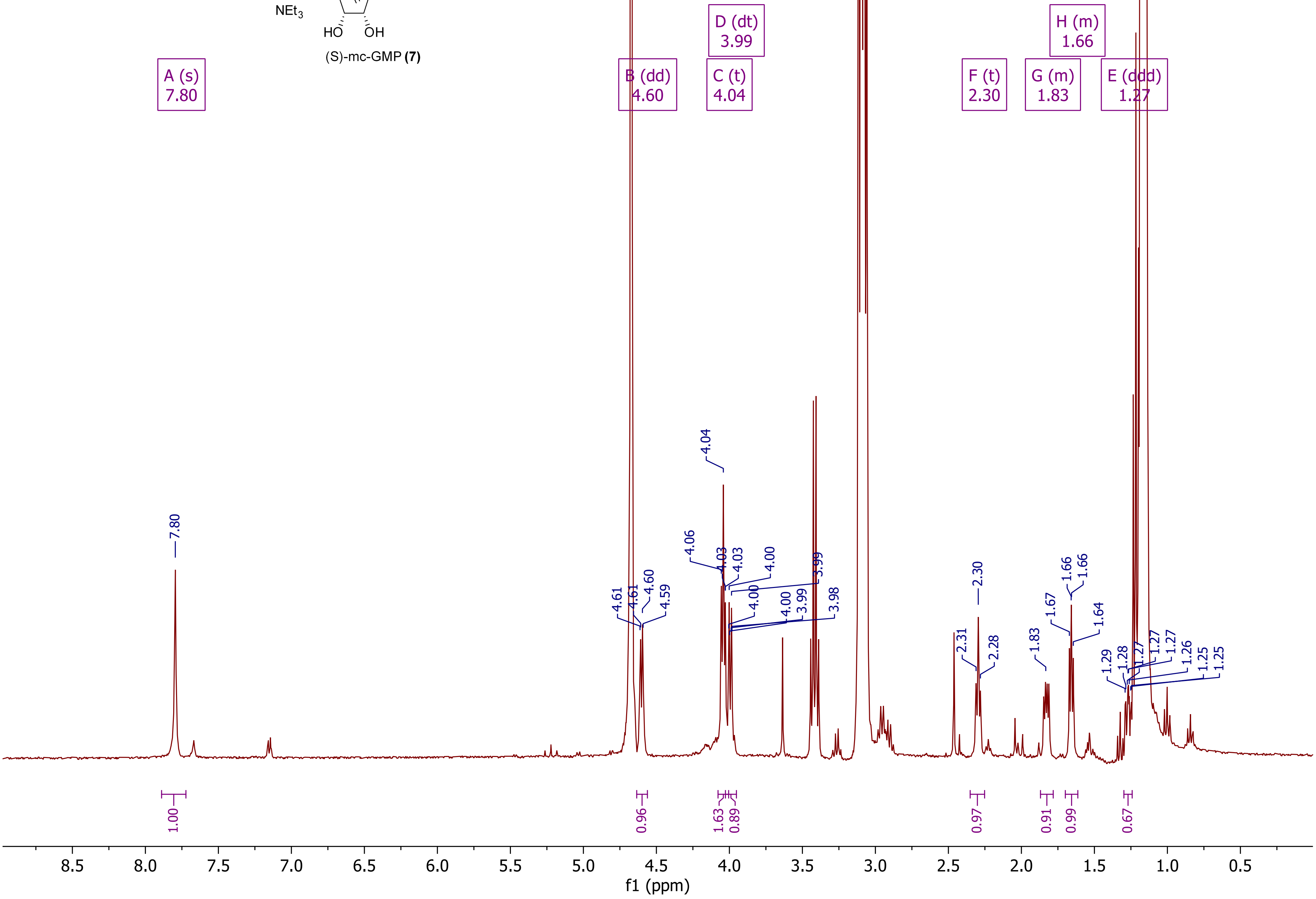

KST3174-p31.1.fid

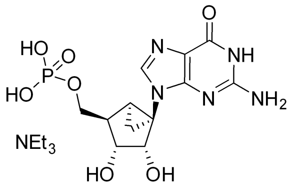

(S)-mc-GMP **(7)**

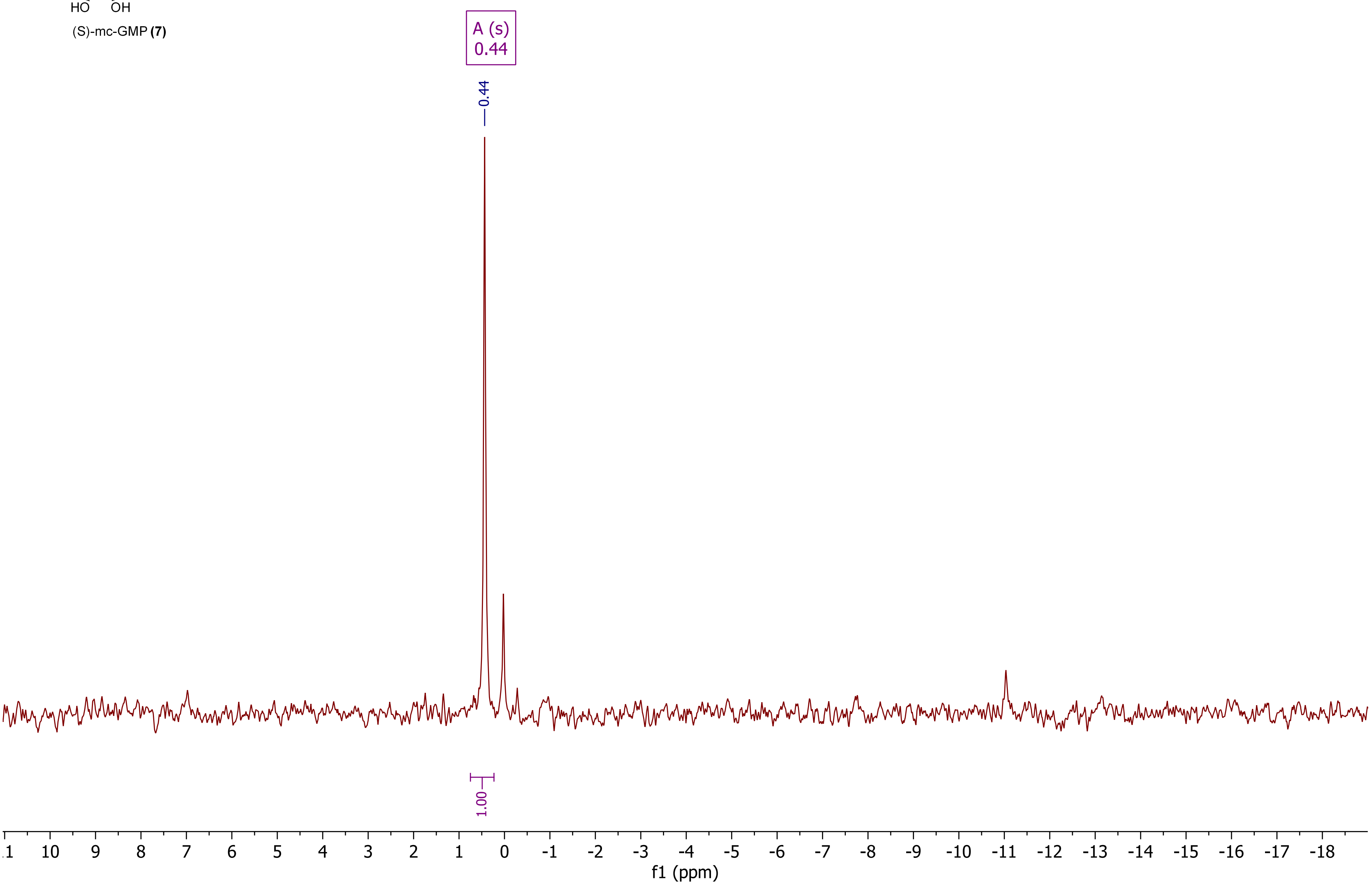

Elemental Composition Report

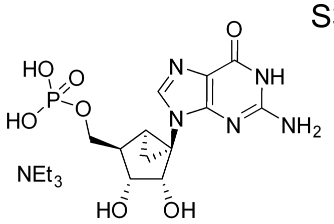

(S)-mc-GMP **(7)**

Single Mass Analysis

Tolerance = 5.0 mDa / DBE: min = -1.5, max = 100.0
Element prediction: Off
Number of isotope peaks used for i-FIT = 3

Monoisotopic Mass, Even Electron Ions
55 formula(e) evaluated with 1 results within limits (up to 50 closest results for each mass)
Elements Used:
C: 0-100 H: 0-200 N: 5-5 O: 0-60 P: 1-1
KST-18MAR19-3-174 66 (1.133) AM2 (Ar,22000.0,0.00,0.00); ABS
TOF MS ES-

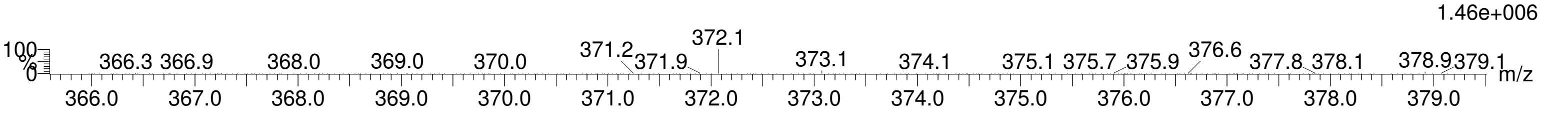

Minimum:				-1.5				
Maximum:		5.0	5.0	100.0				
Mass	Calc. Mass	mDa	PPM	DBE	i-FIT	Norm	Conf(%)	Formula
372.0716	372.0709	0.7	1.9	8.5	343.9	n/a	n/a	C12 H15 N5 O7 P
